# Strengthening primary health care at district-level in Malawi - determining the coverage, costs and benefits of community-directed interventions

**DOI:** 10.1186/s12913-019-4341-5

**Published:** 2019-07-22

**Authors:** Peter Makaula, Mathias Funsanani, Kondwani Chidzammbuyo Mamba, Janelisa Musaya, Paul Bloch

**Affiliations:** 1 Research for Health Environment and Development, P.O. Box 345, Mangochi, Malawi; 2 District Health Office, P.O. Box 42, Mangochi, Malawi; 3 College of Medicine, University of Malawi, Private Bag 360, Chichiri, Blantyre 3, Malawi; 4 Steno Diabetes Center Copenhagen, Niels Steensens Vej 6, NSK 1.11, 2820 Gentofte, Denmark

**Keywords:** Primary health care, Community-directed intervention, Community participation, Essential health service

## Abstract

**Background:**

Community-Directed Interventions (CDI) is a participatory approach for delivery of essential healthcare services at community level. It is based on the values and principles of Primary Health Care (PHC). The CDI approach has been used to improve the delivery of services in areas that have previously applied Community-Directed Treatment with ivermectin (CDTi). Limited knowledge is available about its added value for strengthening PHC services in areas without experience in CDTi. This study aimed to assess how best to use the CDI approach to strengthen locally identified PHC services at district level.

**Methods:**

This was a comparative intervention study carried out over a period of 12 months and involving four health centres and 16 villages assigned to 1) a conventional Essential Health Package (EHP)/PHC approach at health centre level or 2) an EHP/PHC/CDI approach at community level in addition to EHP/PHC at health centre level. Communities decided which intervention components to be included in the intervention. These were home management of malaria (HMM), long lasting insecticide treated nets (LLIN), vitamin A and treatment against schistosomiasis. The outcomes of the two strategies were compared quantitatively after the intervention was completed with regard to intervention component coverage and costs. Qualitative in-depth interviews with involved health professionals, implementers and beneficiaries were carried out to determine the benefits and challenges of applied intervention components.

**Results:**

Implementation of the EHP/PHC/CDI approach at community level as an add-on to EHP/PHC services is feasible and acceptable to health professionals, implementers and beneficiaries. Statistically significant increases were observed in intervention components coverage for LLIN among children under 5 years of age and pregnant women. Increases were also observed for HMM, vitamin A among children under 5 years of age and treatment against schistosomiasis but these increases were not statistically significant. Implementation was more costly in EHP/PHC/CDI areas than in EHP/PHC areas. Highest costs were accrued at health centre level while transport was the most expensive cost driver. The study identified certain critical factors that need to be considered and adapted to local contexts for successful implementation.

**Conclusion:**

The CDI approach is an effective means to increase accessibility of certain vital services at community level thereby strengthening delivery of EHP/PHC services. The approach can therefore complement regular EHP/PHC efforts.

**Trial registration:**

The study was retrospectively registered with the Pan African Clinical Trial Registry TRN: PACTR201903883154921.

**Electronic supplementary material:**

The online version of this article (10.1186/s12913-019-4341-5) contains supplementary material, which is available to authorized users.

## Background

Primary Health Care (PHC) is defined as “*essential health care made universally accessible to individuals and families in the community through their full participation and at a cost that the community and the country can afford to maintain at every stage of their development in the spirit of self-reliance and self-determination*” [1]. PHC was endorsed in 1978 at the Alma Ata, Kazakhstan PHC Conference as a key strategy for attaining equitable access to basic health care, including treatment and prevention of endemic diseases [2]. It is an integral part of health systems in many countries and the first level of contact of individuals, family and community with the public health system. However, as a result of weak health systems PHC implementation remains sub-optimal in Sub-Saharan Africa and access to health services is still a major challenge for a large proportion of the rural population. Weak health systems have also contributed to the persistent high burden of infectious diseases in the rural population [2–5] while hampering effective action against the emerging epidemic of non-communicable diseases. The third goal of the United Nations’ Sustainable Development Goals (SDG) advocates for healthy lives and promotion of well-being for all with the Universal Health Coverage (UHC) being central to it [6]. Both the SDG and UHC are inherently broad, but the PHC approach has the potential to deliver essential health services provided that the existing challenges and gaps [7, 8] are addressed.

Community participation in health programmes enhances their sustainability and affordability [9–11]. However lack of community involvement in most programmes has led to their failure to achieve the desired results [12]. Community-Directed Intervention (CDI) is defined as a health intervention that is undertaken at the community level under the direction of the community itself [13]. The CDI approach has been used successfully to distribute vitamin A and long lasting insecticide treated nets (LLIN) as well as in home management of malaria (HMM) by CDI implementers [13]. A multi-country study conducted in areas where Community-Directed Treatment with ivermectin (CDTi) had previously been implemented demonstrated that CDI is feasible for integrated delivery of different health services in rural Africa [13]. Recently, the CDI approach has also been implemented successfully in areas without prior experience with CDTi [14–18].

Malawi has no explicit PHC policy but implements PHC services through the Essential Health Package (EHP) programme instituted in 2004 [19]. The present study hypothesize that a further extension of the CDI approach to deliver the EHP/PHC services may help fill gaps related to access and delivery in the EHP/PHC system in a rural Malawian setting. The study builds on findings from formative research that was carried out as part of a multi-country study in 2010 [20] showing that intensified community participation based on the CDI approach may be a realistic means to increase accessibility of certain vital interventions at community level in rural Malawian districts, which had no previous experience with CDTi.

As a basis for the planning and implementation of EHP/PHC interventions using the CDI approach in Malawi, the preceding formative research already examined the perceived strengths and weaknesses of existing EHP/PHC related strategies and practices, as well as health providers’ and consumers’ perspectives on PHC in two rural Malawian districts [20]. The present study was therefore implemented in one of the districts, Mangochi, in collaboration with local and national stakeholders already involved in EHP/PHC efforts within the Ministry of Health in Malawi. Specific objectives were:

1. To determine the effects on intervention components coverage of applying the CDI approach as an add-on to the existing EHP/PHC approach for selected public health services in selected villages in Mangochi District.
2. To assess the costs and benefits of applying the CDI approach as an add-on to the existing EHP/PHC approach in Mangochi District.
3. To identify the perceptions of the involved professionals, CDI implementers and beneficiaries about using the CDI approach including the critical factors for its implementation to strengthen EHP/PHC services in Mangochi District.

## Methods

### Study area and setting

**Fig. 1:**
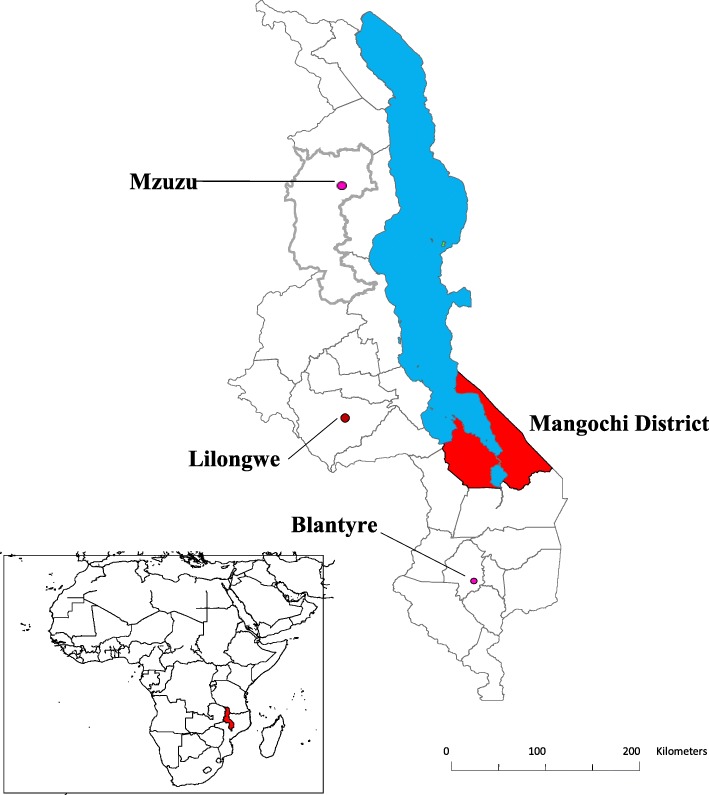
Map of Malawi showing locations of Mangochi District (in red), Lake Malawi (in blue), major cities of Mzuzu, Lilongwe and Blantyre and the location of Malawi in Africa (red in the inset) (Source: Authors’ own map [20])

Malawi is a country in Sub-Saharan Africa with a 2018 population size of 17,563,749 people and 28 districts organized into three regions: northern, central and southern [21]. The study was carried out in Mangochi, one of the 12 districts in the southern region of Malawi. The district is situated on the southern end of Lake Malawi (Fig. 1) and has a total population of 1,148,611 [21]. The district is mainly inhabited by people of Yao and Chewa ethnicities, while Islam (72%) and Christianity (28%) are the most practiced religions. Agriculture, fishing and microbusiness enterprising are the main economic activities of people in the district. Adult literacy is 49%, access to safe water supply is 73%, maternal mortality rate is 400 per 100,000 and the infant mortality rate is 169 per 1,000 [22].

### Study design

The study was designed as a controlled implementation study to run for 1 year. The assumption was that the district received what they needed in terms of information, materials, drugs and other supplies to allow them to satisfactorily implement EHP/PHC services in rural settings and that the study supported the district processes through a CDI based approach. In total, four health centres and 16 villages were involved in the study. Equal numbers of health centres along with their corresponding villages were randomly assigned to either the intervention or control arm of the study (Table 1).

**Table 1:** List of the involved health centres and villages in Mangochi District according to their assigned study arms

District	Health centres	Villages	Assigned study arm
Mangochi	1. Nankumba	1. Saiti Tiputipu 2. Kamangazula 3. Kansiya 4. Binali	A: Intervention - EHP/PHC/CDI approach
	2. Katuli	5. Kwitunji 6. Mponda 7. Sokole 8. Kasanga	
	1. Phirilongwe	1. Makunula 2. Nankamwa 3. Chimwaza 4. Mtendere	B: Control - EHP/PHC approach
	2. Mase	5. Itimu 6. Matenganya 7. Mbalula 8. Meso	

The support rendered included EHP/PHC/CDI training and supervision of district-based health professionals, health centre-based professionals attached to two health centres and community based volunteers/implementers in eight villages in the intervention arm of the study; no such training and supervision support was provided in the two health centres and eight villages involved in the control arm. For the intervention arm, the EHP/PHC/CDI approach was implemented mainly at community level on top of ongoing regular EHP/PHC activities at health centre levels in the same arm thereby complementing rather than replacing the regular health service delivery. In the control arm, the regular EHP/PHC system continued to operate at the health centre level without any EHP/PHC/CDI approach being implemented at community level. By applying these approaches, we expected to assess if CDI could strengthen ongoing EHP/PHC efforts in Mangochi District. Both the EHP/PHC and EHP/PHC/CDI strategies were implemented and evaluated over a period of 12 months.

### Selection of intervention components

The research team identified an intervention package that was agreed with the communities and health care providers during the formative phase based on their priority health issues in the setting [20]. To be properly guided on existing policies regarding the implementation of the EHP/PHC/CDI approach at community level the final choice of intervention components also depended on consultations with key health personnel and other professional stakeholders. The following intervention components were finally included in the study: (i) home management of malaria (HMM) and fever to children under 5 years of age, (ii) distribution of long lasting insecticide treated nets (LLIN) to children under 5 years of age and pregnant women, (iii) vitamin A distribution to children under 5 years of age and (iv) treatment of urinary schistosomiasis using Praziquantel (PZQ) for those above 5 years of age.

### The CDI process and implementation

The study involved a total of five district-based officers consisting of the District Environmental Health Officer, District Community Health Nurse, and three Coordinators for malaria, Neglected Tropical Diseases (NTD) and diarrhoea. These were incorporated into the study as trainers and supervisors after being briefed and trained by the research team on the overall aims of the study, principles and processes of the CDI approach, and on available intervention components of the study. In turn the five district-based officers identified and provided training for two health centre based health workers, who were either a Medical Assistant or a Nurse in charge, and two Senior Health Surveillance Assistants from the two participating health centres under the intervention arm of the study to serve as trainers and later as supervisors for community based CDI implementers. The two health centre based staff in turn engaged their respective villages through community meetings from where 32 community based volunteers (one volunteer per each of the four intervention components in each of the eight intervention villages) were identified, trained and assigned roles as CDI implementers of the selected interventions. At every stage at health centre and community levels, both the research and district teams participated in the trainings as observers to ensure adherence to the study protocol. These health centre based staff later continued to supervise and support CDI implementers in their respective villages throughout the implementation period. No briefing and training were offered out to the staff from the corresponding two health centres and eight villages participating in the control arm of the study. Two implementation partner organizations involved in delivery of health services at district level, Icelandic International Aid Agency (ICEIDA) and Amref Health Africa (AHA) were identified and consulted at the beginning of the study.

During the implementation of the CDI process at community level, the health services, implementation partners and the community played the following roles:

1. Roles of district, health centre staff and implementation partners:
  Identified community leadership structures and socio-cultural organizations, and took these into account in all interactions with the community.
  Introduced to the communities the concept of CDI and technical aspects of the interventions.
  Provided and facilitated capacity building, supplies and technical support as required by the interventions.
  Provided and supported supervision based on procedures and criteria of the intervention.
2. Roles of community members:
  Collectively discussed the health problems and possible intervention components from their own perspective while considering relevant community knowledge and information provided to them by the health professionals.
  Collectively decided whether they will take responsibility for implementation of intervention at community level.
  Collectively designed the approach to implementing the intervention in their communities and identified the required resources from within their respective communities.
  Collectively planned how, when, where and by whom to implement the interventions.
  Collectively supervised and decided on what support to be provided to CDI implementers and how to monitor the processes.
  Executed the intervention (mainly by CDI implementers).
  Collectively reviewed the implementation process where necessary.

### Data collection

The study employed a mixed-method approach to data collection focusing on quantitative data for coverage and cost estimates during baseline and follow-up, and qualitative data for assessing intervention benefits and evaluating processes. Data were collected at district, health centre and village levels in the intervention and control arms of the study. Research assistants collected the necessary data for the study from the involved health professionals, implementation partners, CDI implementers and beneficiaries using 19 data collection instruments previously used by the research group in a 2008 multi-country study [13]. An additional file shows the instruments that were used during data collection [see Additional file 1]. The instruments consisted of survey questionnaires administered to household heads or representatives at community level for determining intervention coverage, and questionnaires administered to relevant health programme coordinators at district level, in-charges at health centres, and leaders at community level, for determining intervention costs. Moreover, Health Management Information System (HMIS) records were reviewed to establish the burden of disease and coverage data at district, health centre and village levels using checklists; Interview guides were used to conduct in-depth interviews with professionals at district and health centre levels, implementation partners at district level and CDI implementers at community level about their perceptions on benefits and critical factors. Finally, focus group discussion guides were used to conduct group interviews with beneficiaries about their perceptions on using the CDI approach. Using these tools data were collected in both intervention and control areas before (baseline) and after (follow-up) introducing the intervention. All the proceedings of the key informant in-depth interviews and focus group discussions were recorded using digital audio recorders. Table 2 summarizes the methods, purposes, sources and quantities of data collected in the study.

**Table 2:** Methods, purposes, sources and amount of data collected in the study

No.	Methods	Purposes	Data collection - phases, levels, arms of study and numbers collected															
			During baseline								During follow-up							
			District	Implementation partners	Intervention			Control			District	Implementation partners	Intervention			Control		
					Health centres	Villages	Households	Health centres	Villages	Households			Health centres	Villages	Households	Health centres	Villages	Households
1.	Questionnaires	Cost	5	2	2	10	20	2	10	20	5	2	2	10	20	2	10	20
		Coverage	–	–	–	–	20	–	–	20	–	–	–	–	20	–	–	20
2.	In-depth interviews	Perceptions/Benefits/Critical factors	–	2	4	16	–	–	–	–	–	2	4	16	–	–	–	–
3.	Focus group discussions	Perceptions/Benefits/Critical factors	–	–	–	4	–	–	–	–	–	–	–	4	–	–	–	–
4.	Health Management and Information System	Coverage/Disease burden	1	2	2	8	–	2	8	–	1	2	2	8	–	2	8	–
5.	Checklist/Observations	Coverage/Disease burden	5	2	2	8	–	2	–	–	5	2	2	8	–	2	–	–
6.	Document reviews	Coverage/Costs/Benefits	1	2	2	–	–	2	–	–	1	2	2	–	–	2	–	–

### Data management and analysis

Quantitative data collected through survey questionnaires and checklists were processed and analyzed using statistical software Epi Info™ version 7.2.1. Analysis involved calculation of percentages, tabulations and frequencies to estimate coverage of individual intervention components. Furthermore, statistical significance tests using Chi Square were performed on differences in delta values (i.e. differences between baseline and follow-up) for each intervention component between intervention and control groups. The analyses of costs and benefits data were carried out using the following procedures:

- The annual total expenditure on each particular recurrent input (e.g. personnel) was calculated for each intervention component.
- Information on the allocation of shared resources was gathered during the field work by asking the persons in charge to indicate what portion of each individual input (recurrent and capital) was used for intervention components in the year (e.g. proportion of time each staff member spent on each CDI related activity). This information was then used to allocate a percentage of the total costs of each individual (recurrent and capital) input to intervention components.
- The opportunity costs were estimated as the loss of productive labour time due to CDI activities. This implied using the proportion of income a volunteer lost when they were involved in CDI activities. Since CDI activities were mainly implemented in rural areas, data on income was not available and the costing analysis therefore used the minimum wage to ascribe a monetary value to free time devoted by the implementers to CDI activities. Implementers’ times were converted and measured in full hours, and this was divided by 8 to give the number equivalent 8-h working days devoted to CDI activities.
- Cost data were converted to United States Dollars at the existing exchange rate at the time the cost was incurred. Then all the costs were inflated to the prevailing United States Dollars by using the prevailing national Consumer Price Index (CPI).

Qualitative data consisted of textual and audio data, including transcripts of key informant in-depth interviews, transcripts of focus group discussions, field notes on observations and other intervention-specific insights, notes and reports from meetings. Transcripts were translated into English and were entered in the computer using standard word processing software. A computer-assisted qualitative content analyses of the data using Atlas-Ti 8, a qualitative data analysis software programme (GmbH 2016) were conducted. Data were analyzed using open coding to come up with cross-classification and retrieval of categories of texts by theme.

## Results

### Characteristics of the study areas

The total population living in the 16 study villages was 20,438 of which 52.4% were from intervention communities and 47.6% were from control communities. Of the total of 3,272 children under 5 years of age and 5,615 women of child bearing age living in the study villages, 47.4 and 44.8% respectively were in the intervention arm of the study. There were 4,511 households in the study villages of which 49.4% were in the intervention arm of the study. Villages in the intervention arm of the study were more distant from their respective health centres, averaging 12.6 km (range 2–35), compared to those in the control arm, averaging 4.9 km (range 2–9). Yao and Islam were the most predominant ethnicity and religion, respectively, in both arms of the study with few exceptions of Chewa and Christian communities in the intervention arm. Table 3 shows the socio-demographic characteristics of the study area.

**Table 3:** Socio-demographic characteristics of the study district, involved health centres and communities

Study arm	Health centre/Village name	Population in crude numbers^a^			Number of households^a^	Approximate distance to the health centre in kilometers	Prominent	
		Total	Under five	Women of child bearing age			Tribe	Religion
	Mangochi District	1,099,666	179,109	242,325	199,110	–	Yao	Islam
A. Intervention: EHP/PHC/CDI approach	1. Nankumba Health centre (22)^b^	27,349	4,649	6,290	4,791	–	Chewa	Christian/Islam
	a) Saiti Tiputipu	2,346	505	539	414	17		
	b) Kamangazula	369	64	75	69	11		
	c) Kansiya	460	90	106	103	24		
	d) Binali	2,279	485	592	433	35		
	2. Katuli Health centre (37)^b^	29,280	4,978	6,734	5,390	–	Yao	Islam
	a) Kwitunji	2,143	107	491	521	2		
	b) Mponda	811	137	186	198	2		
	c) Sokole	431	69	99	65	5		
	d) Kasanga	1,867	93	429	425	5		
B. Control: EHP/PHC approach	3. Phirilongwe Health centre (20)^b^	21,859	3,716	5,028	4,878	–	Yao	Islam
	a) Makunula	2,486	508	520	689	7		
	b) Nankamwa	386	94	88	66	5		
	c) Chimwaza	434	100	100	74	3		
	d) Mtendere	251	60	60	56	9		
	4. Mase Health centre (27)^b^	31,419	5,341	7,226	6,459	–	Yao	Islam
	a) Itimu	1,860	244	893	395	2		
	b) Matenganya	2,761	469	635	586	4		
	c) Mbalula	690	139	388	199	6		
	d) Meso	864	108	414	218	3		

^a^Figures represent the entire catchment population sizes for the mentioned district, health centres and villages

^b^Numbers in brackets represent total villages under each health centre catchment area

According to HMIS data only malaria and fever were among the ten leading causes of morbidity between 2012 and 2016 with malaria in the lead followed by acute respiratory infections (ARI) and skin infections (Table 4). Among these diseases, only malaria and fever were targeted by the four planned intervention components implemented in the present study.

**Table 4:** Leading ten causes of morbidity in Mangochi District from year 2012 up to year 2016

No.	Name of disease/condition causing morbidity	Total cases treated in the district for years			
		2012/13	2013/14	2014/15	2015/16
1.	Malaria 5 years and older	91,279	140,899	143,518	154,294
2.	Malaria under 5 years	106,314	156,284	163,894	162,077
3.	Acute respiratory infections under 5 years	56,254	65,964	76,010	73,449
4.	Skin infection	22,807	30,250	33,882	28,953
5.	Diarrhea non-bloody under 5 years	21,915	23,935	24,312	26,061
6.	Oral condition	12,619	16,203	15,671	16,123
7.	Eye infection	14,694	18,120	16,174	15,849
8.	Common injuries and wounds	14,880	14,665	14,132	14,598
9.	Dysentery	8,823	8,084	11,052	7,504
10.	Sexually transmitted infections (STI)	8,418	8,780	9,854	10,298

(Source: Mangochi Health and Management Information System, 2016)

### The effect of CDI on intervention components coverage

Table 5 presents a summary of the coverage data for each of the four intervention components during baseline and follow-up for both intervention and control arms of the study. The study observed increases in intervention components coverage between baseline and follow-up in all intervention components except distribution of vitamin A among children under 5 years of age in the intervention arm. In the control arm, similar increases in coverage were observed during follow-up in all the intervention components except for HMM and the distribution of vitamin A in children under 5 years of age. The coverage for LLIN distribution at follow-up increased among children under 5 years of age and pregnant women; increases in coverage were further observed for HMM, vitamin A distribution in children under 5 years of age and treatment of urinary schistosomiasis with Praziquantel among those above 5 years of age. Very high coverage rates (above 70%) were registered in LLIN distribution and vitamin A distribution among the children under 5 years of age; followed by a mid-level coverage in LLIN distribution among pregnant women; and very low coverage rates for HMM and urinary schistosomiasis treatment both in the intervention and control arms of the study.

**Table 5:** Estimation of intervention components coverage^a^ for the intervention and control arms of the study during baseline and follow-up

Study arms	Health centre	Number of households	Population^b^			Baseline^c^						Follow-up^c^					
						Presence of LLIN among/in			HMM	Vitamin A	Praziquantel	Presence of LLIN among/in			HMM	Vitamin A	Praziquantel
			Adults	Children	EPW^a^	Households	Children	Pregnant women				Households	Children	Pregnant women			
Intervention	Nankumba	1,019	4,310	1,144	273	934 (91.7%)	1,003 (87.7%)	107 (39.2%)	17 (1.5%)	989 (86.5%)	17 (0.4%)	1,004 (98.5%)	1,141 (99.7%)	100 (36.6%)	40 (3.5%)	1,007 (88%)	173 (4%)
	Katuli	1,209	4,846	406	263	1,048 (86.7%)	358 (88.2%)	141 (53.6%)	17 (4.2%)	349 (86%)	7 (0.1%)	1,173 (97.7%)	293 (92.5%)	216 (82.1%)	2 (0.5%)	324 (79.8%)	63 (1.3%)
	Sub-total	2,228	9,156	1,550	536	1,982 (89%)	1,361 (87.8%)	248 (46.3%)	34 (2.2%)	1,338 (86.3%)	24 (0.3%)	2,177 (97.7%)	1,434 (92.5%)	316 (59%)	42 (2.7%)	1,331 (85.9%)	236 (2.6%)
Control	Phirilongwe	885	2,795	762	178	673 (76%)	548 (71.9%)	79 (44.4%)	175 (23%)	713 (93.6%)	0	883 (99.8%)	665 (87.3%)	103 (57.9%)	180 (23.6%)	726 (95.3%)	11 (0.4%)
	Mase	1,398	5,215	960	309	1,229 (87.9%)	829 (86.4%)	289 (93.5%)	34 (3.5%)	911 (94.9%)	103 (2%)	1,199 (85.8%)	960 (100%)	228 (73.8%)	13 (1.4%)	880 (91.7%)	304 (5.8%)
	Sub-total	2,283	8,010	1,722	487	1,902 (83.3%)	1,377 (80%)	368 (75.6%)	209 (12.1%)	1,624 (94.3%)	103 (1.3%)	2,082 (91.2%)	1,625 (94.4%)	331 (68%)	193 (11.2%)	1,606 (93.3%)	315 (3.9%)

*EPW* Expected number of pregnant women, *HMM* Home management of malaria and fever, *LLIN* Long lasting insecticide treated nets; Praziquantel: For treatment of schistosomiasis

^a^Intervention components coverage data were collected at village level and later lumped up for respective health centres to calculate average figures and percentages

^b^For entire health centres catchment areas

^c^For the four villages involved in the study under each health centre

**Fig. 2:**
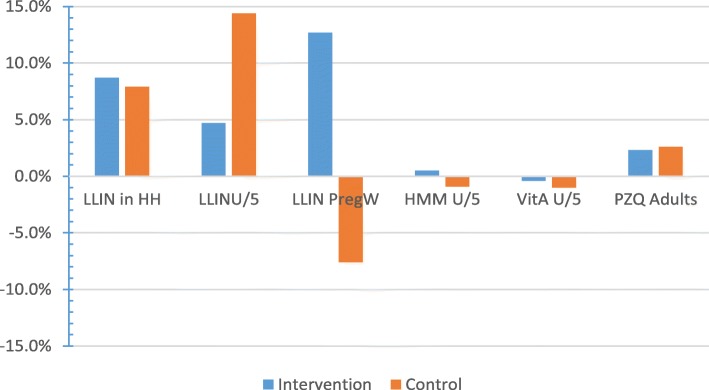
Difference in average coverage rates for intervention and control villages between baseline and follow-up for specific intervention components in the study

Furthermore, when differences (delta values) between baseline and follow-up were calculated for each intervention component in each study arm, it was observed that increases for LLIN among pregnant women and HMM in children under 5 years of age were higher in the intervention arm than in the control arm. For distribution of LLIN and vitamin A in children under 5 years of age, and treatment of urinary schistosomiasis with Praziquantel among individuals above 5 years of age the increases were higher in the control arm than in the intervention arm (Fig. 2).

Chi Square test conducted for each intervention component showed that differences in delta values between intervention and control groups were statistically significant for LLIN distribution in children under 5 years of age (*p*-value 0.003) and pregnant women (*p*-value 0.0075) but not for the remaining four intervention components.

### Assessment of costs, benefits and perceptions of the intervention components

Cost and benefits analyses of the provision of the four intervention components were conducted based on data collected at three levels of study implementation: district, health centre and community. Cost data collected included seven items on opportunity costs of staff salary, volunteer allowances, training, mobilization, transportation, utilities and supervision (Table 6).

**Table 6:** Showing a summary of direct costs of implementing intervention components in the study area

Cost items	Estimated costs according to level and arm used converted to US$^a^					Total US$ for cost item (% of total)
	District	Health centre		Community		
		Intervention	Control	Intervention	Control	
1. Staff salary	138.89	74.09	74.09	–	–	287.07 (3.9)
2. Volunteer allowance	–	–	–	200.00	–	200.00 (2.7)
3. Training	270.84	145.83	–	141.66	–	558.33 (7.6)
4. Mobilization	104.16	83.34	83.34	83.34	–	354.18 (4.8)
5. Transportation	625.01	1,208.34	1,208.34	1,166.67	388.89	4,597.25 (62.7)
6. Utilities	41.67	31.25	31.25	20.84	6.95	131.96 (1.8)
7. Supervision	208.34	166.67	167.67	500.00	166.67	1,209.35 (16.5)
Totals for level/arm (% of total)	1,388.91 (18.9)	1,709.52 (23.3)	1,564.69 (21.3)	2,112.51 (28.8)	562.51 (7.7)	7,338.14 (100)

^a^*US$* United States Dollars

Out of the total amount of resources used to directly implement the selected intervention components at both health centre and community levels most resources were used in the intervention arm of the study. For both arms of the study (intervention and control), the health centre level used more resources than the district level. At community level, only the intervention arm used more resources than the health centre and district levels; the control arm used substantially less resources than the other two levels. According to cost items, transportation costs were the highest across all levels and intervention arms of the study, followed by costs related to supervision and training. For all individual cost items at both health centre and community levels the costs were higher in the intervention arm than the control arm even though a smaller difference in the costs between them was observed at health centre level than at community level.

During follow-up, health workers, implementation partners, CDI implementers and beneficiaries expressed satisfaction with the CDI approach. Most intervention components beneficiaries gave ease to access the services as their main reason for being satisfied. The CDI implementers also expressed satisfaction with their new CDI roles. The satisfaction was derived from the benefits accrued from the intervention components, which was mostly expressed in terms of the tangible improvements experienced by individuals in their own and others’ health, increased availability and accessibility of amenities such as LLIN and drugs, reductions in absenteeism from school or work due to improved health, timely referrals to health facilities, perceived economic benefits or avoidance of hospitalizations associated with higher uptakes of the intervention components.

On the other hand, the health workers and implementation partners perceived the CDI approach as a mechanism to simplify their task of health service provision by empowering the communities which contributed to the reduction of disease burden from the easily manageable and recurrent illnesses such as malaria and anemia. Health workers also indicated reduced workloads on their part because fewer people were being referred to health centres from CDI communities thereby affording health workers more time for other responsibilities.

### Critical factors associated with CDI implementation

Process data according to the involved professionals further identified a number of key factors that positively impacted on the implementation of the CDI approach. These factors were:

1. Engaging stakeholders at all levels to make them understand and appreciate the relevance of the CDI as an alternative mode of delivering health services to people in need and if possible to get them fully or partly on board during the CDI implementation process.
2. Maintaining an enabling and conducive environment for applying the CDI approach in terms of securing supportive policies, availability of supplies, and committed health centre staff.
3. Continuing engaging and empowering communities in efforts to promote local participation and ownership of the CDI implementation process.
4. Selecting appropriate volunteers with requisite skills, willing, trusted and motivated to serve as CDI implementers.

## Discussion

Since most health systems in Sub-Saharan Africa are weak and lack mechanisms of delivering essential health services to those who are most in need, community participation increases service delivery and enhance sustainability [9, 13, 23, 24]. Since the evolution of CDI from CDTi, there has been a series of studies undertaken to assess its effectiveness in areas with prior experience in onchocerciasis control [13]. Later, CDI has also been tested in areas without prior experience with onchocerciasis control with different modifications and settings [18] such as in the control of schistosomiasis and soil transmitted helminths in rural Western Kenya [14] and Mali [15], in delivering multiple health services in urban poor communities in South-western Nigeria [16], and in delivering health services among Fulani Nomads in Enugu State, Nigeria [17]. All these studies have elicited different findings regarding the feasibility of using CDI to deliver essential health services to people in most need in different settings. This study has added to existing knowledge by testing the applicability of CDI at sub district level of the health system, mainly at health centre and community levels in the rural Malawian health district of Mangochi. It has determined the effectiveness of using the CDI approach to deliver the EHP/PHC services to strengthen the EHP/PHC system in Mangochi District and assessed the effects of using the approach on coverage, costs and benefits.

Lack of access to essential PHC services and non-involvement of community in their delivery by healthcare systems are often cited as some of the challenges towards attaining universal health coverage [7–9, 13, 23]. The study showed that the CDI approach of delivering health services to those who are most in need is feasible and acceptable to health professionals, partners, CDI implementers and beneficiaries. The findings revealed that in addition to regular EHP/PHC services the CDI approach can be used to deliver some essential health services in rural areas especially where the health system is not able to serve due to either inadequate personnel or geographical barriers. The coverage estimates in this study has shown that the intervention was effective in the distribution of LLIN to children under 5 years of age and pregnant women. However, the intervention was not effective in relation to the distribution of LLIN in households, home management of malaria, vitamin A distribution among under five children and treatment of schistosomiasis among individuals above 5 years of age because the increases observed during follow-up were not statistically significant. For some intervention components statistically significant decreases in coverage were observed at follow-up. This may be attributed to inadequate supplies for the CDI intervention communities e.g. if there were inadequate LLIN or vitamin A supplied to the communities then coverage of LLIN and vitamin A would not improve no matter the levels of community commitment and competences. Similarly, the treatment of schistosomiasis with Praziquantel was preceded by annual district wide mass drug administration campaign and therefore coverage of Praziquantel treatment may have been affected. These examples question the extent to which the community members (as well as district and health centre based staff) were aware of logistical challenges and whether the communities were empowered to deal with them. The study should have ensured that supplies were adequate (e.g. by contributing supplies during the intervention phase). Another possible explanation that affected coverage relates to distance of individual villages from their respective health centres. On average, intervention villages were located 12.6 km from their respective health centres whereas villages in the control arm were located 4.9 km away from their respective health centres. An example is Binali, one of the villages under the intervention arm, which is located 35 km from its health centre. Differences in geographic distances and high costs of healthcare services is often cited as some of the main contributing factors to disparities in access to essential health services across populations in the world [25, 26]. This disparity in distances between villages and health centres in the two study arms could mean that communities in intervention areas were more disadvantaged in accessing the same intervention components offered in the health centres compared to those in the control areas. One implication of this is that the CDI approach may only be used to complement rather than replace the regular EHP/PHC services offered by the health care system, especially in areas that are located far from health centres. To a large extent these findings are in agreement with the findings of similar studies carried out elsewhere [13–18] and showing that the CDI approach is effective in increasing service coverage and accessibility but is vulnerable to distance and cost factors as shown in this study. When direct costs of implementing CDI were computed into the analysis it revealed that the EHP/PHC/CDI services required almost twice as many financial resources compared to regular EHP/PHC services. Although this finding was not predictable, the higher costs are mainly due to transport, supervision and training, which are also vital for the success of CDI implementation. This high cost of implementing the CDI approach may be considered a disincentive in short term but as an investment, it may become cheaper in the long term due to the cumulative effect of disability-adjusted life years gained through resultant improved health services or due to the building up of community capacity to handle their own health challenges. The higher costs are also a consequence of a greater number of participants involved in the intervention arm of the study. The inclusion of high coverage intervention components may thus justify the high cost observed due to the implementation of the CDI approach.

The costs of indirect leveraged contributions related to administrative, logistical, personnel, supplies, drugs, LLIN, infrastructure and technical expertise made by participating institutions (i.e. Ministry of Health, University of Malawi College of Medicine, Research for Health Environment and Development and Steno Diabetes Center Copenhagen) towards implementation of the project have not been included in this analysis but are estimated to have covered 75% of the overall costs of the project. If these indirect contributions were to be included in the determination of the overall costs then it would not be cost effective to use the CDI approach to strengthen delivery of essential PHC services in the District. However, the low coverage obtained in some of the intervention components coupled with the limited effects observed for some of the health services may point to the existence of inherent weaknesses in the selection of intervention components and unforeseeable factors as discussed above. There is therefore a need to re-examine the reasons and factors that may be attributed to the low coverage and limited effects of some of the intervention components as revealed in this study.

For optimum implementation of the CDI approach, the study documented the processes, critical factors, barriers and enablers which can influence either way towards its outcome. It is therefore important when planning similar studies within Malawi or elsewhere, to adhere to the implementation processes and take into consideration all the critical factors described in this paper.

The study revealed that beneficiaries, CDI implementers and health workers were satisfied with the implementation of CDI due to perceived benefits accrued from the implementation of the targeted intervention components. The benefits mentioned associate with easiness to access health services, increased availability of vital amenities, improvements in their health, reduction in absenteeism from school or work, timely referrals to health facilities, avoidance of hospitalizations, reduction of disease burden and reduced workloads in health facilities. These sentiments and perceptions are similar to those also expressed in other studies within the Sub-Sahara African region [9, 13–18] and reinforce the need to incorporate the CDI approach to strengthen the delivery of PHC in Sub-Saharan Africa where scarcity of resources in the health sector prevail.

Some barriers that can affect the implementation process were also identified and documented. They include the prevailing shortage of health personnel in district health facilities across Malawi. This intensifies work pressure of available health personnel who find it difficult to manage daily duties, including those related to CDI. Other factors were related to motivation of the involved health staff and volunteers to appreciate the importance of community involvement. These motivational factors were mostly linked to material or monetary expectations due to inadequate remuneration, and provision for food and transport while occupied with CDI related work. Existence of a supportive government policy framework for community-based delivery of interventions is an enabler for implementation of the CDI approach. To this effect, the Malawi Government is applauded for promulgating two distinct and important policy documents in the National Community Health Strategy and the Health Sector Strategic Plan II, which guarantees commitment and political will from the health systems towards community empowerment through initiatives such as CDI [27, 28]. Another enabler is the existence of a network of community-based volunteers and health workers who are actively involved in the delivery of various health services at community level in Malawi. This is a valuable resource that can be exploited in favour of the CDI approach.

A major weakness of the study is that it assumed that supplies to the intervention and the control arms were similar and adequate, i.e. if supplies differed then this would affect coverage rates immediately even if all other factors such as well-trained participants, high commitment and engagement, professional delivery of essential health services at community level etc. were adequate. This assumption therefore was not fulfilled since allocation and prioritization of resources within the health systems (at district and health centre levels) were beyond the study’s control. This has an implication to our interpretation of the study’s findings because if the assumption fails then it becomes very difficult to answer if CDI as an add-on to EHP/PHC is effective or not. Another weakness in the present study is that despite mainly being implemented in villages with different population sizes, most of our analysis and presentation have lumped the villages together with uniform sample sizes and focused more on the health centres. Embracing the characteristics of individual study villages would have increased the richness of our findings. However, as a minimum the study has made it clear that coverage and cost data were collected in the involved villages even though the data were later lumped together, calculated and presented as health centre averages and percentages.

Important future research areas relate to assessment of resources availability at health centre level and prioritization processes for how and where these resources were used (e.g. at health centre or community level), to test applicability of the CDI approach to improve access to essential health services in exclusively hard to reach rural or peri-urban areas, as Malawi endeavors to attain UHC [27, 28] and also to conduct a cost-benefit analysis for using the CDI approach to strengthen PHC delivery at sub-district level in order to inform policy formulation in Malawi or elsewhere.

## Conclusions

This study has demonstrated that implementation of the CDI approach in rural areas of Mangochi District is feasible, acceptable and effective. In terms of intervention components coverage, it was effective for increasing LLIN among children under 5 years of age and pregnant women. It was however not effective for increasing coverage of distribution of vitamin A and home management of malaria among children under 5 years of age and treatment against schistosomiasis in those above 5 years age. Faced with acute shortage of trained health workers in many healthcare systems in low-income countries, the approach can be considered as an add-on for the delivery of selected essential health services in rural areas. It should be used to complement the regular EHP/PHC services offered by the healthcare system especially in areas where personnel and geographical barriers exist. For maximum outcomes, the design and implementation of intervention components have to take into consideration certain critical factors and should be adapted to suit with local conditions and contexts.

## Additional file

Additional file 1: Instruments that were used during data collection for the study consisting of survey questionnaires administered to various respondents, checklists for health facility records, interview guides for key informants and focus group discussion guides with the beneficiaries. (DOCX 99 kb)

## Data Availability

The datasets used and/or analyzed during the current study are available from the corresponding author on reasonable request.

## Abbreviations

AHA: Amref Health Africa
ARI: Acute respiratory infections
CDI: Community-directed intervention
CDTi: Community-directed treatment with ivermectin
EHP: Essential health package
EPW: Expected number of pregnant women
GmbH: *Gesellschaft mit beschränkter Haftung* (Company with limited liability)
HMIS: Health Management Information System
HMM: Home management of malaria and fever
ICEIDA: Icelandic International Development Agency
LLIN: Long lasting insecticide treated net
NHSREC: National health sciences research ethics committee
NTD: Neglected tropical diseases
PHC: Primary Health Care
PregW: Pregnant women
PZQ: Praziquantel
SDG: Sustainable Development Goal
UHC: Universal Health Coverage
US$: United States Dollars
Vit A: Vitamin A
