# Supplementary material for: Strengthening primary health care at district-level in Malawi - determining the coverage, costs and benefits of community-directed interventions
Source: BMC Health Serv Res. 2019 Jul 22;19:509. doi: 10.1186/s12913-019-4341-5 (PMC6647329; doi:10.1186/s12913-019-4341-5)
Supplement: Supplementary file 1 — Instruments that were used during data collection for the study consisting of survey questionnaires administered to various respondents, checklists for health facility records, interview guides for key informants and focus group discussion guides with the beneficiaries. (DOCX 99 kb) [file 12913_2019_4341_MOESM1_ESM.docx]

**STRENGTHENING PRIMARY HEALTH CARE IN RURAL MALAWIAN DISTRICT USING THE COMMUNITY-DIRECTED INTERVENTION APPROACH**

1. **CHECKLIST FOR MINUTES OF MEETINGS**

During all CDI meetings with health officials, partners, village leaders and communities, the researcher present should keep detailed notes and prepare detailed minutes of the meeting. The following is the checklist of process issues that should be incorporated in these minutes in addition to a summary of the main discussion and conclusions.

**Checklist of process issues that need to be incorporated into recording the minutes of meetings**

1. Meeting venue, date and time
2. List of attendees, occupation and addresses
3. Focus of meeting
4. The agenda if any
5. Who chairs the meeting? Elected or self-imposed. Issues discussed
6. How discussion was done (who contributes – all or some?
7. Who dominates the discussion and who are silent?
8. Are there some interest groups who try to impose their views?
9. Are all members who want to contribute given the chance?
10. Non-verbal reactions
11. How decisions on each item were reached – Consensus or by an individual or group of cliques?
12. Are there protests to decisions reached and by whom?
13. Whether the issues and decisions are summarized and presented to members
14. Reaction of members to summarized presentation when meeting ended
15. Post meeting comments heard from people
16. Collect papers circulated to members during the meeting
17. **INFORMATION AND PROCEDURE FOR ELLICITING CONSENT**

**Risk and benefits to participants**

Subjects are at no risk of physical harm when participating in the evaluation of the project. The potential for social risk is negligible, if any. Participants will benefit from the intervention and providers, members of the community and direct beneficiaries will be interviewed and/or observed in order to obtain the required information on how. Participants will not be required to discuss any topics with which they do not feel comfortable.

Participants will be given an opportunity to talk about their experience and be heard. For many this will be first. The eventual objective is to develop a strategy for community-directed intervention against diseases that are endemic in the communities, which is of immense benefit to the study groups and the wider population of people living in the area.

**Informed consent**

All consent forms (I-III) will be pilot tested for comprehensibility and modified if necessary, prior to the start of the study. The written consent forms will be translated into the local languages of *Chichewa* and *Chiyao* and back-translated into English to ensure accuracy before use, and signed by the participant before interviewing. If the participant is illiterate, the consent form will be read to them in their native language and verbal consent documented on the form by a witness not associated with the study.

**Procedure for obtaining informed consent**

*Participants in the in-depth interviews, focus groups and observation groups*

The interviewer and/or the study coordinator will give a copy of the consent form to the interviewee for the interviews (if literate) and read out loud by the facilitator and/or the study coordinator (in a group discussion) to ensure that the group clearly understands what is being asked of them. Participants that have questions regarding the project will be given a copy of the form for their records. Further the Principal Investigator will make available the project fact sheet for the records of the community.

*People who may appear in pictures describing actions or lifestyle*

In some cases pictures will be taken describe some activities. The team member who is responsible for taking the pictures will be required to ask these people at the beginning of the study for their collective informed consent to take certain types of photographs. Team members in the project will approach people they would like to photograph, introduce them, before taking the photos. If the people are willing to have their photographs taken, they will be asked in the presence of other people to say so.

**Compensation for time and costs**

*Compensation:* As much as possible non-monetary compensation will be used in the study to an equivalent value of $2 each. Compensation will be in form of a present (which will be of domestic value such as a basin, childcare appliance for women and for men some other culturally appropriate material) as reimbursement for their time and effort when participating in the interviews, focus groups and observations.

*Potential costs of participation:* Participants will not incur any financial costs for being in the study. The only cost of their participation is the time they allow for answering the interview and survey questions.

**Confidentiality of the data**

All the data collected through the interviews, focus group discussions, questionnaires and observations will be kept confidential. Participants will be assured of confidentiality in the research effort and confidentiality amongst any group will be stated as a requirement in the consent form. Participants will also be asked to give their permission to have some of their photos and text (chosen by participants) displayed in a public setting and/or in an academic manual to describe illness experiences research and health education. To protect the identities of the participants, true names will not be used. Participants will give signed consent to participate and their signatures will be kept in a locked filing cabinet.

**Public release of data**

Data gleaned from this project may also be used to inform an academic manual that describes approaches for using photography in the health sciences and/or community based research. Photos and text will only be used after obtaining permission from the participants.

**Overall intent of the research effort**

This study will allow us to better understand the factors that influence the direct participation of community members in intervening against diseases that affect them. Recommendations will be suggested for the researchers and health providers initiating national and local programmes for the management of diseases of different complexity.

**What is the purpose of this project?**

The purpose of this study is to collect information on the factors that influence community participation and the experiences of community members, community directed service providers and direct beneficiaries of interventions that are directed by the community in order to determine how Primary Health Care in rural Malawi can be strengthened using the Community-Directed Intervention approach. This study will explore and allow us to better understand, through interviews, and observations, of the extent to which community can participate in the intervention against diseases that affect them.

**What area of health is the project interested?**

Project staff is interested in how the community in this area, with the facilitation of the formal health service/system, will participate in the intervention/s against major health problems as identified by the communities such as malaria, vitamin A deficiency and others.

**How will project staff gather information?**

Project staff will gather information through interviews, discussions and observations.

**How will the findings from this project be used?**

Findings may be used in professional journal articles, exhibits, books, reports and educational manuals. Recommendations will be derived from this study for the global Primary Health Care system researchers in developing countries initiating national and local programmes for the management of tropical diseases with the direct participation of affected communities.

**What is important for communities/individuals who would participate in the project to know?**

All data collected through the in-depth interviews, discussions, survey questionnaires, and during observation sessions will be kept confidential. Communities and individuals will be asked to sign informed consent to assure that they understand the purpose of the project, what the project is about, and that they can choose to withdraw participation at any time.

*Form I*

**Strengthening Primary Health Care in rural Malawian districts using the Community-Directed Intervention approach**

## Informed Consent - Interviews (individuals and community)

This project we want you to join is a study. There are many organizations that would like to have the communities participating in interventions against diseases that affect them so as to reduce the time and cost of intervention. The Ministry of Health and its partners are collecting information on people’s experiences while directing intervention at the community level. We will ask questions about many aspects of life in this community (household/family). We will ask about how your people decided on the approach to use for interventions against the diseases and the roles individuals and the health sector played. We want to learn more about your experience with the intervention activities. We may take pictures of activities in the community, which will help us to explain our findings. We will go to different households, talk with women, men and like you in this community (household) either in groups or individually. Some of the conversation may be taped so that we do not miss out some of the important things that are said.

If you choose to be in this study, we will ask you questions about your personal experiences. The questions are general but if you find that some questions are not going well with you, please do not feel compelled to answer any of them for any reason. We will talk to you for about 45-60 minutes. You can decide if you want to take part in this study. Taking part in this study will not cost you anything. You may also leave the study at any time. You can leave for any reason without any problems. You and your family may not get any direct benefits from being in the study. What you tell us will help us better develop a strategy for training community members to manage malaria at home, onchocerciasis, tuberculosis, and vitamin A deficiency and thus improve the health of the people in this community. Your name and what you say to us for this study will be kept private as much as the law allows.

Do you have any questions about the study? If you have any questions about your rights in the study, you may contact Mr Peter Makaula at Research for Health Environment and Development (RHED), Mangochi at +265 888 850 829 during the study and in the future. If you have other questions about the study, you may also contact the project coordinator Mr Peter Makaula at RHED, Mangochi during the study and in the future. If you have concerns about human rights, ethics and welfare issues contact the National Health Sciences Research Committee (NHSRC) of the Ministry of Health at PO Box 30377, Lilongwe 3, Malawi.

If you agree to answer our questions, you can tell us that you agree by repeating these words and then putting your name and signature in the space below.

*I have read the foregoing information, or it has been read to me. I have had the opportunity to ask questions about it and any questions I have been asked have been answered to my satisfaction. I consent voluntarily to participate as a subject in this study and understand that I have the right to withdraw from the study at any time without in any way affecting my further medical care.*

__________________________________________________ _______________

Community Leader/Individual Participant’s name/signature Date

_________________________________________________ _______________

Interpreter/Witness’s Signature Date

*Form II*

**Strengthening Primary Health Care in rural Malawian districts using the Community-Directed Intervention approach**

**Informed Consent – Interview for coverage (individuals)**

This project we want you to join is a study. There are many organizations that would like to have the communities participating in interventions against diseases that affect them so as to ensure that as many people as possible benefit from it. The Ministry of Health and its partners are collecting information on people’s experiences about types of services received from people who were selected by members of your community (CDI Implementers) to provide health services for community members in the management of malaria and vitamin A deficiency. We will talk about the types of services that were received, in what forms and amount from different types of implementers, whether the treatments are effective, the type of side effects experienced and what was done to deal with them. For those who did not receive services, we will ask them why they did not receive services and what needs to be done for them to receive missed services if they desire them. We want to learn more about your experience with these intervention activities. We will go to different households, talk with women, men and children like you in this community (household) individually.

If you choose to be in this study, we will ask you questions about your personal experiences. We may also request to see the treatment card that the implementers gave to you or other members of your household in the course of his/her work. The questions that we will ask you are general but if you find that some questions are not going well with you, please do not feel compelled to answer any of them for any reason. We will talk to you for about 10 minutes. You can decide if you want to take part in this study. Taking part in this study will not cost you or your family anything. You may also leave the study at any time. You can leave for any reason without any problems. You and your family may not get any direct benefits from being in this study. What you tell us will help us better develop a strategy for training community members to manage malaria at home and vitamin A deficiency and thus improve the health of the people in this community. Your name and what you say to us for this study will be kept private as much as the law allows.

Do you have any questions about the study? If you have any questions about your rights in the study, you may contact Mr Peter Makaula at Research for Health Environment and Development (RHED), Mangochi at +265 888 850 829 during the study and in the future. If you have other questions about the study, you may also contact the project coordinator Mr Peter Makaula at RHED, Mangochi during the study and in the future. If you have concerns about human rights, ethics and welfare issues contact the National Health Sciences Research Committee (NHSRC) of the Ministry of Health at PO Box 30377, Lilongwe 3, Malawi.

If you agree to answer our questions, you can tell us that you agree by repeating these words and then putting your name and signature in the space below.

*I have read the foregoing information, or it has been read to me. I have had the opportunity to ask questions about it and any questions I have been asked have been answered to my satisfaction. I consent voluntary to participate as a subject in this study and understand that I have the right to withdraw from the study at any time without in any way affecting my further medical care.*

_______________________________________________ _______________

Individual Participant’s name/signature Date

_______________________________________________ ________________

Interpreter/Witness’s signature (verbal consent) Date

*Form III*

**Strengthening Primary Health Care in rural Malawian districts using the Community-Directed Intervention approach**

**Informed Consent – Interview with Health Workers (individuals)**

This project we want you to join is a study. There are many organizations that would like to have the communities participating in interventions against diseases that affect them so as to improve its coverage and reduce the time and cost of intervention. The Ministry of Health and partners are collecting information on the experiences of health workers about the community directed interventions at the community level. We will ask questions about your attitude to the community directed interventions and types of support given. We will also ask about what types of services that were provided by you and other team members before during and after the programme. We want to learn more about your experiences with these intervention activities. We will go to different health care workers; talk with female and male staff in health care facilities or at the district level either in groups or individually. Some of the conversations may be taped so that we do not miss out some of the important things that are said.

If you choose to be in this study, we will ask you questions about your personal experiences. We will also observe you while carrying out some of your work. The questions that we will ask you are general but if you find that some questions are not going well with you, please do not feel compelled to answer any of them for any reason. We will talk to you for about 45-60 minutes. You can decide if you want to take part in this study and will not cost you and your family anything. You may also leave the study at any time. You can leave for any reason without any problems. You and your family may not get any direct benefits from being in this study. What you tell us will help us better develop a strategy for training community members to manage malaria at home and vitamin A deficiency and thus improve the health of the people in the community. Your name and what you say to us for this study will be kept private as much as the law allows.

Do you have any questions about the study? If you have any questions about your rights in the study, you may contact Mr Peter Makaula at Research for Health Environment and Development (RHED), Mangochi at +265 888 850 829 during the study and in the future. If you have other questions about the study, you may also contact the project coordinator Mr Peter Makaula at RHED, Mangochi during the study and in the future. If you have concerns about human rights, ethics and welfare issues contact the National Health Sciences Research Committee (NHSRC) of the Ministry of Health at PO Box 30377, Lilongwe 3, Malawi.

If you agree to answer our questions, you can tell us that you agree by repeating these words and then putting your name and signature in the space below.

*I have read the foregoing information, or it has been read to me. I have had the opportunity to ask questions about it and any questions I have been asked have been answered to my satisfaction. I consent voluntary to participate as a subject in this study and understand that I have the right to withdraw from the study at any time without in any way affecting my further medical care.*

_______________________________________________ _______________

Individual Participant’s name/signature Date

_______________________________________________ ________________

Interpreter/Witness’s signature (verbal consent) Date

1. **LIST OF RESEARCH INSTRUMENTS AND THEIR ADMINISTRATION**

| **Instrument** | | **To be administered to** | **Timing** |
| --- | --- | --- | --- |
| 1 | Survey questionnaire for LLIN coverage | Household head or other persons who may provide required information in 5 sample households with under 5 children in each of the 8 sample evaluation villages in each cluster. Information to be collected on every household member. | End of year |
| 2 | Survey questionnaire for HMM coverage | Women/child minders with children less than 5 years old in 5 sample households with under 5 children in each of the 8 sample evaluation villages in each cluster. | End of year |
| 3 | Survey questionnaire for vitamin A coverage | Women/child minders with children less than 5 years old in 5 sample households with under 5 children in each of the 8 sample evaluation villages in each cluster. | 1 month after last delivery |
| 4 | Survey questionnaire for praziquantel coverage | Every member aged above 5 years of 5 selected sample households in each of the 8 evaluation villages in each district | End of year |
| 5 | Factor questionnaire for programme coordinators | Programme manager for each intervention at district level. | End of year |
| 6 | Factor questionnaire for programme leaders | 1 traditional leader; 1 woman leader; and 2 community development leaders, CBO leader or religious group leader in each of the 10 sample evaluation villages in each cluster | End of year |
| 7 | Checklist for community level | Traditional leader assisted by community agents in each of the 10 sample evaluation villages in each cluster | End of year |
| 8 | Checklist for district level | Programme manager in each district | End of year |
| 9 | Checklist for NGO level | 1 focal person for each NGO involved in delivery of intervention in study areas | End of year |
| 10 | In-depth interview for CDI implementers | 1 male and 1 female implementer in each village; 1 male and 1 female formal health workers per village | End of year |
| 11 | In-depth interview for partners | 1 focal person per NGO, donor, MoH, UN agencies, CBOs and other relevant community groups in each cluster | End of year |
| 12 | Community level focus group discussion (FGD) | 2 youth male FGDs, 2 youth female FGDs, 2 adult male FGDs and 2 adult female FGDs per cluster | End of year |
| 13 | Background costing date sheet | Information to be obtained for relevant documents | End of year |
| 14 | Cost questionnaire for programme coordinators | Programme coordinators for the 5 interventions at district and national levels | End of year |
| 15 | Cost questionnaire for in-charge at district level | Officer in-charge at district level | End of year |
| 16 | Cost questionnaire for first line health facility | Officer in-charge at first line health facility | End of year |
| 17 | Cost questionnaire for community leaders | Community leaders/key informant in 10 evaluation villages in each cluster | End of year |
| 18 | Cost questionnaire for community volunteers | Volunteers for the study interventions in 10 evaluation villages in each cluster. | End of year |
| 19 | Cost questionnaire for households | Head of households of 5 sample households in each of 10 evaluation villages in each cluster | End of year |

1. **RESEARCH INSTRUMENTS FOR COVERAGE**

| **Instructions for data collection using the instruments for coverage**  Respondent selection from the Household   - A modified EPI sampling strategy will be used in the community. - Locate a central point, for example, the church, the market on the chief’s house (face the central point). - Turning the back to the central point (such as the chief’s house), throw a pencil to select the direction to proceed. - For example the researcher commences from the right-hand side if the pencil falls with tip otherwise the researcher will commence from the left. - Once the direction is known, the researcher will enter each household to interview eligible respondents until the number of the required households is obtained. |
| --- |

1. **RESEARCH INSTRUMENTS FOR PROCESS AND FACTORS**

**FORM 1:** SEMI-STRUCTURED QUESTIONNAIRE FOR DISTRICT HEALTH PROGRAMME OFFICERS/COORDINATORS

**INSTRUCTIONS:**

1. **Who to interview:** Programme managers for each of the study interventions in the study district. Note that study districts include those in both the intervention and control arms in the entire study. This is the same wherever it is encountered in the instruments.
2. **Sample:** Select 1 programme manager per intervention in each study district.
3. **When:** Administer instrument before and after intervention

**Country: ______________________ [__] District: ________________ [__]**

**Identification Code: [__][__][__][__][__] Enumerator: ______________ Date: __/__/__**

The Community-directed Intervention intends to use the Community for delivery of health interventions (LLINs, Home Management of Malaria, Vitamin A and schistosomiasis). In this context, the Community, the Health Services and other partners have specific sites to perform in order to ensure the integration of these interventions.

Please would you like to take part and provide information to the following questions? Information provided will be kept strictly confidential.

[If yes, proceed with interview. If no, stop]

**Name** (optional): ______________________ **Sex**: 1=Male [__] 2=Female [__]

**Number of years of experience**: __________ **Designation**: ______________________

**Name of intervention**: _________________________________

1. **Awareness/Perception**

1. When was CDI introduced in this community?

Probe into:

(a) The people who brought the idea about CDI process

(b) Whether there was a community meeting at that time

(c) Number of people selected and trained to be community direct implementers

2. How is the CDI process performing in this community?

Probe into:

(a) Number of CDI still functioning

(b) Method of incentive for the CDI

(c) **How adequate are the incentives given to CDI?**

3. Please describe how the community took the decision of implementing the CDI process?

Probe for the following issues:

(a) People involved in the decision making

(b) How the people in the community came to know the decision

(c) Method and time of distribution of drugs/items

(d) Have they participated and approved the CDI selection?

(e) Was the incentive of the CDI a participatory decision?

4. What are the likely consequences of giving gifts (such as money, materials like bicycles, food items, farm crops) to community people who are involved in the management of diseases in the community? ______________________________________________

_____________________________________________________________________

_____________________________________________________________________

**B. Health worker availability to support CDI process**

5. Are health workers available to provide support to the CDI process?

1=Always available [__]→ GOTO Q7

2=Sometimes available [__]

3=Never [__]

6. How can such a situation be improved? _____________________________________

_____________________________________________________________________

_____________________________________________________________________

7. Do they (Health Workers) experience any difficulties in meeting up with the demands of the CDI process?

1=Yes [__]

2=No [__]→ (Stop)

8. What are these difficulties? ______________________________________________

9. How can these difficulties be overcome? ____________________________________

**C. Assessing the interventions**

10. What are the benefits of the CDI interventions? (Probe for economic and social benefits)

11. What are the costs implications for CDI interventions? (Probe for economic and social and opportunity costs)

12. What can you say about the complexity levels of the interventions? Which ones were easier for the community and which ones were difficult?

**D. Health workers attitude towards CDI process**

Some health workers often make some statements about the CDI process. Kindly indicate your agreement/disagreement with the following statements.

|  | **Statements** | Strongly  agree | Agree | Indifference | Disagree | Strongly  disagree |
| --- | --- | --- | --- | --- | --- | --- |
| 1 | Because there are few health services in the communities, community members have to take some health care responsibilities. |  |  |  |  |  |
| 2 | The performance of CDI in this community/area is satisfactory. |  |  |  |  |  |
| 3 | Distribution of drugs like Vitamin A, praziquantel is best done by health workers. |  |  |  |  |  |
| 4 | Communities are not capable of organizing and monitoring home treatment of uncomplicated malaria. |  |  |  |  |  |
| 5 | The involvement of community members in health activities allows for frequent monitoring and supervision by health staff. |  |  |  |  |  |
| 6 | Community members should not handle malaria drugs because they are not trained health workers. |  |  |  |  |  |
| 7 | The involvement of community members in health and development activities enhances health services in this community. |  |  |  |  |  |
| 8 | Community members are quite capable of supervising tuberculosis treatment during the continuation phase. |  |  |  |  |  |
| 9 | There are many problems with the performance of CDI in this community/area. |  |  |  |  |  |
| 10 | Community involvement in drug distribution saves the time of the health worker to do other things. |  |  |  |  |  |
| 11 | Community involvement in malaria treatment is a take-over of the duties of the health worker. |  |  |  |  |  |

**COMMENTS AND SUGGESTIONS (for community involvement in health activities):**

**___________________________________________________________________________**

**___________________________________________________________________________**

**___________________________________________________________________________**

**___________________________________________________________________________**

**___________________________________________________________________________**

**___________________________________________________________________________**

**___________________________________________________________________________**

**FORM 2: SEMI-STRUCTURED QUESTIONNAIRE FOR COMMUNITY LEADERS**

**INSTRUCTIONS:**

1. **Who to interview:** Community leaders. These include:

- Traditional leader
- Women leader
- Community development group leader
- Major CBO leader
- Religious group leaders (both orthodox and traditional)

1. **Sample:** Select the traditional leaders, one women leader and one each from any other two groupings in the community. This brings to four the number of community leaders to be interviewed.
2. **When:** Administer instrument before and after intervention

**Country: ______________________ [__] District: ________________ [__]**

**Village Name: _____________________ [__] [__] [__] Household ID No. [__][__]**

**Identification Code: [__][__][__][__][__] Enumerator: ______________ Date: __/__/__**

The Community-directed Intervention intends to use the Community for delivery of health interventions (LLINs, Home Management of Malaria, Vitamin A and Praziquantel). In this context, the Community, the Health Services and other partners have specific sites to perform in order to ensure the integration of these interventions.

Please would you like to take part and provide information to the following questions? Information provided will be kept strictly confidential.

[If yes, proceed with interview. If no, Stop]

**Position in the Community:** ______________________ **Name (Optional): _____________**

**Number of years as Community leader:** _____________ **Occupation: ________________**

**Sex:** 1=Male [__] 2=Female [__]

1. **Awareness/Perception**
2. When was CDI introduced in this community?

Probe into:

(a) The people who brought the idea about CDI process

(b) Whether there was a community meeting at that time

(c) Number of people selected and trained to be community direct implementers

1. How is the CDI process performing in this community?

Probe into:

(a) Activities carried out in the past year.

(b) Number of CDI still functioning.

(c) Method of incentive for the CDI?

3. Please describe how the community took the decision of implementing the CDI process during the last year. (Note that CDI decision-making should occur throughout the year, not just at the inception of the programme.)

Be sure that mention is made of specific decisions and then probe into the following issues.

(a) People involved in the decision making

(b) How the people in the community came to know the decision

(c) Method and time of distribution of drugs (for the different interventions),

(d) Have they participated and approved the CDI selection?

(e) Was the incentive of the CDI a participatory decision? How?

(f) What types of incentives are given to CDIs by this community?

(g) How adequate are these incentives for the CDIs?

4. What are the likely consequences of giving gifts (such as money, materials like bicycles, food items, farm crops) to community people who are involved in the management of diseases in the community? ______________________________________________

____________________________________________________________________

**B. Health worker availability to support CDI process**

1. Are health workers available to provide support to the CDI process?

1=Always available [__]→ GOTO Q3

2=Sometimes available [__]

3=Never [__]

2. How can such a situation be improved? _____________________________________

_____________________________________________________________________

3. Do they (Health Workers) experience any difficulties in meeting up with the demands of the CDI process?

1=Yes [__]

2=No [__]→ **[If No in Q3, Stop section B and move to section C below]**

4. What are these difficulties? ______________________________________________

5. How can these difficulties be overcome? ____________________________________

_____________________________________________________________________

**C. Assessing the interventions**

6. What are the benefits of the CDI interventions? (Probe for economic and social benefits) ____________________________________________________________________

____________________________________________________________________

7. What are the costs implications for CDI interventions? (Probe for economic and social and opportunity costs) __________________________________________________

_____________________________________________________________________

8. What can you say about the complexity levels of the interventions? Which ones were easier for the community and which ones were difficult? _______________________

_____________________________________________________________________

**D. Community members’ attitude towards CDI process**

Indicate your strength of agreement/disagreement with the following statements about the CDI process.

|  | **Statements** | Strongly  agree | Agree | Indifference | Disagree | Strongly  disagree |
| --- | --- | --- | --- | --- | --- | --- |
| 1 | Because there are few health services in the communities, community members have to take some health care responsibilities. |  |  |  |  |  |
| 2 | The performance of CDI in this community/area is satisfactory. |  |  |  |  |  |
| 3 | Distribution of drugs like Vitamin A, Praziquantel is best done by health workers. |  |  |  |  |  |
| 4 | Communities are not capable of organizing and monitoring home treatment of uncomplicated malaria. |  |  |  |  |  |
| 5 | The involvement of community members in health activities allows for frequent monitoring and supervision by health staff. |  |  |  |  |  |
| 6 | Community members should not handle malaria drugs because they are not trained health workers. |  |  |  |  |  |
| 7 | The involvement of community members in health and development activities enhances health services in this community. |  |  |  |  |  |
| 8 | Community members are quite capable of supervising tuberculosis treatment during the continuation phase. |  |  |  |  |  |
| 9 | There are many problems with the performance of CDI in this community/area. |  |  |  |  |  |
| 10 | Community involvement in drug distribution saves the time of the health worker to do other things. |  |  |  |  |  |
| 11 | Community involvement in malaria treatment is a take-over of the duties of the health worker. |  |  |  |  |  |

**COMMENTS AND SUGGESTIONS (for community involvement in health activities):**

**___________________________________________________________________________**

**___________________________________________________________________________**

**___________________________________________________________________________**

**___________________________________________________________________________**

**___________________________________________________________________________**

**___________________________________________________________________________**

**___________________________________________________________________________**

**FORM 3: CHECKLIST FOR INTERVIEW AND OBSERVATION OF CRITICAL FACTORS FOR DELIVERY OF VITAMIN A, LLIN, HOME MANAGEMENT OF MALARIA AND SCHISTOSOMIASIS AT COMMUNITY LEVEL**

**INSTRUCTIONS:**

1. **Who to interview:** Traditional leader to be assisted by community agent
2. **Sample:** Select 1 respondent per community.
3. **When:** Administer instrument before and after intervention

**Project Site: ______________________ [__] District: ________________ [__]**

**Village Name: _____________________ [__] [__] [__] Household ID No. [__][__]**

**Identification Code: [__][__][__][__][__] Enumerator: ______________ Date: __/__/__**

The Community-directed Intervention intends to use the Community for delivery of health interventions (LLINs, Home Management of Malaria, Vitamin A and Praziquantel). In this context, the Community, the Health Services and other partners have specific sites to perform in order to ensure the integration of these interventions.

Please would you like to take part and provide information to the following questions? Information provided will be kept strictly confidential.

[If yes, proceed with interview. If no, Stop]

**1. Census (Tick as appropriate)**

|  | By Interview | | By Observation | | Comments |
| --- | --- | --- | --- | --- | --- |
|  | Yes | No | Yes | No |  |
| Is there a record on community population census? |  |  |  |  |  |

**2. Keeping of records**

| How many implementers (CDI or other type) keep records on | <50% | 50-75% | >75% | Comments |
| --- | --- | --- | --- | --- |
| Vitamin A supplementation |  |  |  |  |
| LLIN |  |  |  |  |
| Home management of malaria |  |  |  |  |
| Treatment of schistosomiasis |  |  |  |  |

**3. Completeness of records (Tick as appropriate)**

|  | # Complete | # Not complete | Comments |
| --- | --- | --- | --- |
| How many of the records assessed are complete (kept by implementers) |  |  |  |
| Vitamin A (age) |  |  |  |
| LLIN (# treated, # retreated) |  |  |  |
| Home management of malaria (age, sex, dosage) |  |  |  |
| Treatment of schistosomiasis (age, sex, dosage) |  |  |  |

**FORM 4: CHECKLIST FOR INTERVIEW AND OBSERVATION OF CRITICAL FACTORS FOR DELIVERY OF VITAMIN A, LLIN, HOME MANAGEMENT OF MALARIA AND SCHISTOSOMIASIS AT DISTRICT LEVEL**

**INSTRUCTIONS:**

1. **Who to interview:** Programme managers/coordinators for each of the intervention programmes in the study district
2. **Sample:** Select 1 programme manager/coordinator for each programme.
3. **When:** Administer instrument before and after intervention

**Project Site: ______________________ [__] District: ________________ [__]**

**Identification Code: [__][__][__][__][__] Enumerator: ______________ Date: __/__/__**

The Community-directed Intervention intends to use the Community for delivery of health interventions (LLINs, Home Management of Malaria, Vitamin A and schistosomiasis). In this context, the Community, the Health Services and other partners have specific sites to perform in order to ensure the integration of these interventions.

Please would you like to take part and provide information to the following questions? Information provided will be kept strictly confidential.

[If yes, proceed with interview. If no, Stop]

**1. Existence of supporting policy. (Tick as appropriate)**

|  | By Interview | | By Observation | | Comments |
| --- | --- | --- | --- | --- | --- |
|  | Yes | No | Yes | No |  |
| Is there a written policy to support to support CDI process for |  |  |  |  |  |
| Vitamin A supplementation |  |  |  |  |  |
| LLIN |  |  |  |  |  |
| Home management of malaria |  |  |  |  |  |
| Treatment of schistosomiasis |  |  |  |  |  |

**2. Availability of drugs or products.**

| State # of designated centres for which drugs or products are available? | # Available | # Not available | Comments |
| --- | --- | --- | --- |
| Vitamin A supplementation |  |  |  |
| LLIN |  |  |  |
| Home management of malaria |  |  |  |
| Treatment of schistosomiasis |  |  |  |

**3. Availability of transport**

| Is there functioning means of transport for CDI supervision? | By Interview | | By Observation | | Comments |
| --- | --- | --- | --- | --- | --- |
|  | Yes | No | Yes | No |  |
|  |  |  |  | |  |

**4. Budget for transport for supervision**

| Is there a current budget for transportation for supervision of: | By Interview | | By Observation | | Comments |
| --- | --- | --- | --- | --- | --- |
|  | Yes | No | Yes | No |  |
| Vitamin A supplementation |  |  |  |  |  |
| LLIN |  |  |  |  |  |
| Home management of malaria |  |  |  |  |  |
| Treatment of schistosomiasis |  |  |  |  |  |

**5. Availability of supervision team**

| Is there a supervision team at district level for: | By Interview | | By Observation | | Comments |
| --- | --- | --- | --- | --- | --- |
|  | Yes | No | Yes | No |  |
| Vitamin A supplementation |  |  |  |  |  |
| LLIN |  |  |  |  |  |
| Home management of malaria |  |  |  |  |  |
| Treatment of schistosomiasis |  |  |  |  |  |

**6. Availability of storage space**

| How many of your health facilities have space for storage of commodities for: | By Interview | | By Observation | | Comments |
| --- | --- | --- | --- | --- | --- |
|  | # with space | # without space | # with space | # without space |  |
| Vitamin A supplementation |  |  |  |  |  |
| LLIN |  |  |  |  |  |
| Home management of malaria |  |  |  |  |  |
| Treatment of schistosomiasis |  |  |  |  |  |

**7. Incorporation of CDI in District Implementation Plans**

| Does the 2012 district health plan incorporate CDI process for: | By Interview | | By Observation | | Comments |
| --- | --- | --- | --- | --- | --- |
|  | Yes | No | Yes | No |  |
| Vitamin A supplementation |  |  |  |  |  |
| LLIN |  |  |  |  |  |
| Home management of malaria |  |  |  |  |  |
| Treatment of schistosomiasis |  |  |  |  |  |

**8. Availability of budget for CDI process**

| Is there a budget for CDI process in 2003 for: | By Interview | | By Observation | | Comments |
| --- | --- | --- | --- | --- | --- |
|  | Yes | No | Yes | No |  |
| Vitamin A supplementation |  |  |  |  |  |
| LLIN |  |  |  |  |  |
| Home management of malaria |  |  |  |  |  |
| Treatment of schistosomiasis |  |  |  |  |  |

**9. Availability of a register on stock management**

| Is there a register on stock management for: | By Interview | | By Observation | | Comments |
| --- | --- | --- | --- | --- | --- |
|  | Yes | No | Yes | No |  |
| Vitamin A supplementation |  |  |  |  |  |
| LLIN |  |  |  |  |  |
| Home management of malaria |  |  |  |  |  |
| Treatment of schistosomiasis |  |  |  |  |  |

**10. Reporting for CDI**

| For how many facilities in year 2003 did you receive report/returns for: | <50% | 50-75% | >75% | <50% | 50-75% | >75% | Comments |
| --- | --- | --- | --- | --- | --- | --- | --- |
| Vitamin A supplementation |  |  |  |  |  |  |  |
| LLIN |  |  |  |  |  |  |  |
| Home management of malaria |  |  |  |  |  |  |  |
| Treatment of schistosomiasis |  |  |  |  |  |  |  |

**FORM 5: CHECKLIST FOR INTERVIEW AND OBSERVATION OF CRITICAL FACTORS FOR DELIVERY OF VITAMIN A, LLIN, HOME MANAGEMENT OF MALARIA AND SCHISTOSOMIASIS (PARTNERSHIP STRUCTURES E.G. NGOs, DONORS, MINISTRY OF HEALTH ETC).**

**INSTRUCTIONS:**

1. **Who to interview:** All focal persons for NGOs, Donor agencies, MOH and UN agencies operating with the study Districts.
2. **Sample:** Select 1 focal person for each group (NGO, Donor agencies, MOH and UN agencies) in the study Districts.
3. **When:** Administer instrument before and after intervention

**Country: ______________________ [__] District: ________________ [__]**

**Identification Code: [__][__][__][__][__] Enumerator: ______________ Date: __/__/__**

The Community-directed Intervention intends to use the Community as it is used for delivery of health interventions (LLINs, Home Management of Malaria, Vitamin A and Praziquantel). In this context, the Community, the Health Services and other partners have specific sites to perform in order to ensure the integration of these interventions.

Please would you like to take part and provide information to the following questions? Information provided will be kept strictly confidential.

[If yes, proceed with interview. If no, Stop]

Fill in the boxes

|  | By Interview | | By Observation | Comments |
| --- | --- | --- | --- | --- |
|  | NGOs | Donors | Local partners |  |
| 1. How many partners are involved in CDI process at district level? |  |  |  |  |
| Vitamin A supplementation |  |  |  |  |
| LLIN |  |  |  |  |
| Home management of malaria |  |  |  |  |
| Treatment of schistosomiasis |  |  |  |  |

|  | By Interview | | By Observation | Comments |
| --- | --- | --- | --- | --- |
|  | NGOs | Donors | Local partners |  |
| 2. How many are involved in routine planning and budgeting for? |  |  |  |  |
| Vitamin A supplementation |  |  |  |  |
| LLIN |  |  |  |  |
| Home management of malaria |  |  |  |  |
| Treatment of schistosomiasis |  |  |  |  |

|  | By Interview | | By Observation | Comments |
| --- | --- | --- | --- | --- |
|  | NGOs | Donors | Local partners |  |
| 3. How many provide direct funding for CDI for? |  |  |  |  |
| Vitamin A supplementation |  |  |  |  |
| LLIN |  |  |  |  |
| Home management of malaria |  |  |  |  |
| Treatment of schistosomiasis |  |  |  |  |

|  | By Interview | | By Observation | Comments |
| --- | --- | --- | --- | --- |
|  | NGOs | Donors | Local partners |  |
| 4. How many provide logistic support (transport, commodities) for CDI for? |  |  |  |  |
| Vitamin A supplementation |  |  |  |  |
| LLIN |  |  |  |  |
| Home management of malaria |  |  |  |  |
| Treatment of schistosomiasis |  |  |  |  |

|  | By Interview | | By Observation | Comments |
| --- | --- | --- | --- | --- |
|  | NGOs | Donors | Local partners |  |
| 5. How many provide training for CDI for? |  |  |  |  |
| Vitamin A supplementation |  |  |  |  |
| LLIN |  |  |  |  |
| Home management of malaria |  |  |  |  |
| Treatment of schistosomiasis |  |  |  |  |

|  | By Interview | | By Observation | Comments |
| --- | --- | --- | --- | --- |
|  | NGOs | Donors | Local partners |  |
| 6. How many provide support for advocacy for CDI for? |  |  |  |  |
| Vitamin A supplementation |  |  |  |  |
| LLIN |  |  |  |  |
| Home management of malaria |  |  |  |  |
| Treatment of schistosomiasis |  |  |  |  |

**FORM 6: IN-DEPTH INTERVIEW GUIDE FOR CDI IMPLEMENTERS (E.G. CDI IMPLEMENTERS AT INTERVENTION COMMUNITIES AND FORMAL HEALTH WORKERS)**

**INSTRUCTIONS:**

1. **Who to interview:** CDI Implementers in intervention communities and formal health workers in both intervention and control Districts.
2. **Sample:**

a. Interview one CDI Implementers in each of the 8 sample communities in the district. Determine in advance if female CDI Implementers are available and purposely sample up to 5, but not two from the same community. If more than 5 communities have a female CDI Implementers, ballot for just five commodities. Interview male CDI Implementers in the remaining communities.

b. Identify the front line health facilities that serve the 8 sample communities. These should be the facilities where community members go to collect their drugs and other supplies prior to distribution and where they submit treatment records. Ballot for (2) Health centre and interview the officer in charge or another senior person who is available.

1. **When:** Administer instrument before and after intervention

***CDI Implementers’ willingness to perform multiple CDI tasks***

**Country: ______________________ [__] District: ________________ [__]**

**Village/community: _______________**

**Position: ______________ CDI Implementer/ Health Worker Sex: ______  Female  Male**

**Introduction:**

Good day. I am ……………… … I am from Ministry of Health and wish to learn about how you feel about participating in the delivery of Community-directed interventions (CDI) such as Vitamin A distribution, home management of malaria (HMM), long lasting insecticide treated nets (LLIN) and schistosomiasis treatment. We will ask you questions about your personal experience. The questions are general but if you find that some questions are not well with you, please do not feel compelled to answer any of them for any reason. We will talk to you for about 10 - 15 minutes. Participation in this interview is voluntary and you may choose to terminate the interview if you so decide without any repercussion. What you tell us will help us develop a strategy for training community members to manage the interventions stated earlier and thus improve the health of the people. Do you have any questions about the study? If you have any questions about your rights in the study or in case of emergency, you may contact Mr Peter Makaula of RHED – Malawi on +265 888 850 829 or Mr Edwin Nkhono of Ministry of Health on +265 888 895 073.

Are you willing to participate? Yes [__] No [__]

**If No, Thank respondent and terminate interview.**

1. a) What are the health activities you have been involved in this community?
2. How did you get involved in CDI activities?
3. When did you get involved?

*Probe for a) and b)*

1. Vitamin A
2. Home management of malaria
3. LLIN
4. Schistosomiasis treatment
5. What motivates you to work on several interventions together?
6. How willing are you to carry out these activities? Give reasons?
7. In what ways are you being motivated in carrying out CDI activities? Probe for motivation from (a) the community and (b) the government.

*Probe for type and adequacy of motivation for:*

1. Vitamin A
2. Home management of malaria
3. LLIN
4. Schistosomiasis treatment
5. What motivates you to work on several interventions together?
6. Has it ever happened that you felt like abandoning your tasks as a CDI implementer?

Why?

1. How much time do you spend on CDI activities in a month?

*Probe as appropriate*

1. Vitamin A
2. Home management of malaria
3. LLIN
4. Schistosomiasis treatment
5. What difficulties do you face when carrying out these activities?

*Probe as appropriate*

1. Vitamin A
2. Home management of malaria
3. LLIN
4. Schistosomiasis treatment
5. (Also ask about difficulties with integration-make some suggestions.)
6. What recommendations can you make for improving delivery of CDI activities?

*Probe for:*

1. Vitamin A
2. Home management of malaria
3. LLIN
4. Schistosomiasis treatment
5. (Also ask about recommendations for integrating activities…)

**Assessing the interventions**

1. What are the benefits of the CDI interventions?

*Probe for economic and social benefits*

1. What are the costs implications for CDI interventions?

*Probe for economic, social and opportunity costs*

1. What can you say about the complexity levels of the interventions? Which ones were easier for the community to implement and which ones were difficult?

Name of respondent: __________________ Name of Interviewer: ___________________

**FORM 7: IN-DEPTH INTERVIEW GUIDE FOR PARTNERS**

**INSTRUCTIONS:**

1. **Who to interview:** All focal persons for NGOs, Donor agencies, MOH and UN agencies operating with the study Districts as well as CBOs and community groups.
2. **Sample:** Select 1 focal person for each group (NGO, Donor agencies, MOH and UN agencies) in the study Districts as well as the CBOs and community groups.
3. **When:** Administer instrument before and after intervention

**Country: ______________________ [__] District: ________________ [__]**

**Type of organisation: NGO [__] CBO [__] Community Group [__] Date: __/__/__**

**Introduction:**

Good day. I am ……………… … I am from Ministry of Health and wish to learn about how you feel about participating in the delivery of Community-directed interventions (CDI) such as Vitamin A distribution, home management of malaria (HMM), long lasting insecticide treated nets (LLIN) and schistosomiasis treatment. We will ask you questions about your personal experience. The questions are general but if you find that some questions are not well with you, please do not feel compelled to answer any of them for any reason. We will talk to you for about 10 - 15 minutes. Participation in this interview is voluntary and you may choose to terminate the interview if you so decide without any repercussion. What you tell us will help us develop a strategy for training community members to manage the interventions stated earlier and thus improve the health of the people. Do you have any questions about the study? If you have any questions about your rights in the study or in case of emergency, you may contact Mr Peter Makaula of RHED – Malawi on +265 888 850 829 or Mr Edwin Nkhono of Ministry of Health on +265 888 895 073.

Are you willing to participate? Yes [__] No [__]

**If No, Thank respondent and terminate interview.**

**Section A: Perception of partners towards CDI process**

1. How did you get involved in the CDI activities and when?
2. Why did your organization decide to get involved in CDI activities?
3. What role does your organization play in the partnership?
4. Does your organization experience any problems in the partnerships?
5. If yes, which ones?
6. If no, what makes for success or participation by your organization?

**Section B: Special arrangements for dialogue, decision-making and problem solving.**

1. Has your organization put any **arrangements** for dialogue and problem solving within the partnership?
2. If yes, what are these **arrangements**?
3. How do they function?
4. How have these arrangements influenced the CDI process? Probe for positive and negative impacts.

**Section C: Assessing the interventions**

1. What are the benefits of the CDI interventions? (Probe for economic and social benefits)
2. What are the costs implications for CDI interventions? (Probe for economic, social and opportunity costs)
3. What can you say about the complexity levels of the interventions? Which ones were easier for the community to implement and which one were difficult?

Name of respondent: __________________ Name of interviewer: ____________________

**FORM 8: FOCUS GROUP DISCUSSION GUIDE FOR COMMUNITY MEMBERS (YOUTH FEMALE, YOUTH MALE, ADULT FEMALE AND ADULT MALE)**

***PLEASE NOTE:*** *(1) Each sub-group should have between 5-8 members.*

*(2) Conduct 2 FGDs for each sub-group per study site*

**INSTRUCTIONS:**

1. **Who to interview:**

a. Youth male

b. Youth female

c. Adult male

d. Adult female

1. **Sample:** 2 groups of 5-8 persons for each category (youth male, youth female, adult male and adult female) per study site, i.e. District.
2. **When:** Administer instrument before and after intervention

**Introduction**

Good day, I am ________________ and my colleagues are _________________ from the Ministry of Health and are here to learn from you about some type of activities related to the delivery of Community-directed interventions such as Vitamin A distribution, Home management of malaria (HMM), long lasting insecticide treated nets (LLIN) and schistosomiasis treatment that are going on in this community. We have invited you because of your experience in this community and the confidence we have in you.

Please note that in this discussion there are no right and wrong answers. Every opinion is important and should be freely expressed. What we will learn from you today will be useful in the future to implement CDI activities.

We will talk to you for about 45-60 minutes. Participation in this interview is voluntary and you may choose to terminate the interview if you decide without any repercussion. What you tell us will help us develop a strategy for training community members to manage the interventions stated earlier and thus improve the health of the people in this community. Your name and what you say to us for this study will be kept private. We wish to request your permission to take notes and tape record the conversation so as to ensure that we do not miss out or misrepresent any of your views after the discussion.

Do you have any questions about the study? In case you do have questions in the course of the discussion or afterwards, please do not hesitate to contact Mr Peter Makaula of RHED-Malawi on +265 888 850 829.

Are you willing to participate? Yes [__] No [__]

**If no, thank participants and terminate discussion.**

**General information**

1. What are the health facilities in this community?

Probe into:

- Availability of health care facilities and how they function in the community
- Who owns the facilities
- Who manages them
- Who uses the facilities
- Nearness of the facilities
- How well are these health care facilities satisfying the need of this community
- Existence of VHWs and type of activities they perform and at what cost

1. What types of diseases do you experience in this community?

Probe for:

- Most common diseases experienced in this community
- The most serious and severe of these diseases (malaria, blindness due to vitamin A deficiency) to the community members?
- Relationship of community members to the following diseases: malaria, blindness?
- Reasons for such relationships.

**Community ownership of the CDI process and decision-making**

Introduction:

*The experiences of using health workers in managing and controlling diseases that exist in African countries are costly and services may not be easily available. It has been shown that since these diseases are within the community, therefore, the community could take charge of managing and controlling these diseases through active participation. Community directed interventions (CDI) strategy has been successful and we would like to see if this approach could be used in the treatment and control of malaria and the distribution of Vitamin A*.

1. When was CDI introduced in this community?

Probe into:

- The people who brought the idea about CDI process
- Whether there was a community meeting at the time
- Number of people selected and trained to be community directed implementers
- How did CDI implementation/CDI implementer selection relate to existing health activities?

1. How is the CDI process performing in this community?

Probe into:

- Number of CDI implementers still functioning (if some of the CDI implementers are no longer functioning, probe for reasons why?)
- Method of incentive for the CDI?
- Ask about the mix of interventions – how working together, how satisfied?

1. Please describe how the community took the decision of implementing the CDI process during the past year? (Note that CDI is ongoing).

Elicit specific decisions/actions and then probe to the following issues:

- People involved in the decision making
- How the people in the community came to know the decisions?
- Method and time of distribution of drugs
- Have they participated and approved the CDI selection?
- How does the community reward CDI implementers? Probe for the decision process for incentives, if any?

1. In what ways can members of the community support the CDI process?

**End of the discussion; please thank the participants for their time and participation.**

**FORM 9: RESEARCH INSTRUMENTS FOR COSTING**

**Costing data collection guide***

This section describes the instruments for collecting data for costing of the interventions. Costing data will be collected at three levels: (i) the institutional level comprising of the formal health institutions in the study districts (district health offices and first line health facilities) and the corresponding national and regional offices, (ii) the community level and (iii) the household level. Both financial and economic costs will be collected. Financial costs entail actual intervention expenditure; and economic costs include opportunity costs such as value of donated goods and services, which are easily estimated by taking their equivalent market prices. Community volunteer labour will be estimated by finding out whether the volunteers receive a salary or wages elsewhere and use that to cost their donated time. Otherwise an equivalent value based on the national minimum wage structure will be used to estimate the cost of the time donated by the volunteer. This is, all resources consumed will be valued.

1. **Capital Input Costs** (*Capital inputs are defined as inputs that last for more than one year*):
2. Buildings – Space: administrative offices, hospitals, health centres, training rooms, storage facilities, (baseline only)
3. Equipment: computers, printers, refrigerators, sterilizers, manufacturing machinery, scales, flip charts, video, audio, megaphones, billboards, nets, and other equipment with unit cost (price) of US$100 or more.
4. Vehicles: 4-wheel drive vehicles, trucks, motor cycles and bicycles.
5. Training – non recurrent: Training activities for health personnel or community members that occur only once or rarely.
6. Social mobilization – non recurrent: Social mobilization activities that occur only once or rarely.
7. Consultancies – non recurrent.
8. Other capital input costs not included in above.
9. **Recurrent Input Costs** *(Recurrent inputs are resources that are consumed or replaced within one year. Although recurrent inputs often made up of items of individually small value that are complements to capital inputs and they are usually borne by recipient health districts, health areas and communities in many public health-related programmes, they are frequently purchased in large amounts and thus can contribute significantly to overall costs)*.
10. Personnel (all types): Managers, physicians, nurses, supervisors, health workers, administrators, technicians, consultants, implementers, CDI Implementers, community leaders, drivers, casual labour.
11. Supplies and miscellaneous: Drugs, insecticides, vaccines, syringes, stationeries, consumables, diskettes, tapes, refreshment, leaflets, dipping basins, sachets, rent, other small equipment with unit cost (price) under US$100.
12. Buildings – operation and maintenance: electricity, water, heating, fuel, telephone, fax, telex, insurance, cleaning, printing, repairs of electricity, plumbing, roofing.
13. Vehicles – operation and maintenance: petrol, diesel, lubricants, tyres, spares, registration, insurance, taxes.
14. Logistics/Transport – Travel: all logistic items and local transport and travel fees within and between districts by taxi, bus, motorcycle or bicycle.
15. Training – Recurrent: short retraining, in service courses, training workshop or seminars.
16. Social mobilization – Recurrent: operational costs for screening and involvement of different stakeholders or partners.
17. Monitoring and Evaluation/Consultancies – Recurrent
18. Other operating costs not included in above.

**Collection of costing data during the study**

Some cost components are sensitive to the changes that will be introduces through CDI during the study period, while others such as the costs for buildings, are not likely to be affected during the relatively short study period of 12 months. Hence the baseline data will be used for costing capital items and are unlikely to change during the study period, while at the end of the year of the study, the focus of the data collection will be on the relevant recurrent cost items such as personnel time (opportunity costs for volunteer time, salaries for district health staff), use of vehicles, training costs, drugs and LLINs etc.).

The collection of costing data during the study will use the data collection forms and procedures as described in this study protocol developed by economists during a workshop held in May 2006 in Entebbe, Uganda.

| **FORM 10: BACKGROUND COSTING DATA** |
| --- |

*(To be collected from published data from Country Statistics Data Agencies or International Financial Statistics of the IMF). Where local rates (e.g. local inflation rate, local interest rate) must apply, these should be obtained from the former source.*

Country: ____________________________________________________________

Intervention: _________________________________________________________

Local currency: _______________________________________________________

Discount rate (for base year): _____________________ Source: _____________

Consumer Price Indices (CPI) for years spanned by data collection

Year: ______________ CPI: ________________

______________ ________________

Market Exchange Rates for years spanned by data collection

Year: ____________ Local Currency: ___________ US$: ____________

____________ ___________ ____________

Official Exchange Rates for years spanned by data collection

Year: ____________ Local Currency: ___________ US$: ____________

____________ ___________ ____________

Local Market Interest Rates for years spanned by data collection

Year: ______________ Interest Rates: ________________

______________ ________________

Local Official Interest Rates for years spanned by data collection

Year: ______________ Interest Rates: ________________

______________ ________________

Local Inflation Rates for years spanned by data collection

Year: ______________ Inflation Rates: ________________

______________ ________________

**Cost at the institutional level**

Procedures for collecting recurrent costs at the health institutional level.

Questionnaire is to be administered to each of the programme managers for the study interventions (HMM, LLIN, Vitamin A, Schistosomiasis) at the institutional level. One questionnaire should be completed for each study intervention in each study district.

Questionnaire on drug and LLIN supplies and should be administered to the officer in-charge of the health institution.

| **FORM 11: COST AT INSTITUTIONAL LEVEL AT END OF THE STUDY YEAR** |
| --- |

**PROJECT CODE [__] DISTRICT CODE [__] DATE _ _/_ _/_ _ _ _**

**EVALUATION [__]** (0=Baseline 1=Evaluation)

**INTERVIEW OF PROGRAMME MANAGER FOR: (25 per site) plus 5 at the Regional level.**

Intervention [__] (1=HMM, 2=LLIN, 3=Vitamin A, 4=Schistosomiasis)

1. **Recurrent Personnel Costs**

1.1 Can you tell me how man personnel are involved in this intervention?

1. Number of salaried employees? [__][__]
2. Number of volunteers at the level of your institution? [__][__]
3. Number of consultants? [__][__]

*Please include all personnel involved, whether they work for the government or for other organizations, e.g. NGOs (but do not include community level volunteers)*

1.2 Could you please assist to complete the following tables by listing for all personnel mentioned above (employees and volunteers) their grade, monthly gross salary, monthly allowance + benefits, the source of funding and times allocated on the intervention?

**Table 1.2.c. Consultants**

| **Consultants** | **Type of consultancy**  1. Management.  2. Public health specialist,  3. Other technical expertise,  4. Transport,  5. Others, specify | **Annual fees and allowances** | **Funded by** *(code as many as relevant)*  1. Community,  2. Individuals,  3. Local NGO,  4. International NGO,  5. Government,  6. UN agencies |
| --- | --- | --- | --- |
|  |  |  |  |
|  |  |  |  |
|  |  |  |  |
|  |  |  |  |
|  |  |  |  |
|  |  |  |  |
|  |  |  |  |
|  |  |  |  |
|  |  |  |  |
|  |  |  |  |

**2. Recurrent Training Costs:** procedures for collecting data on training costs at the health institutional level**.**

2.1 Did you conduct any training session for this intervention? [__] (1=Yes; 2=No)

If yes, how many training sessions did you conduct during the last 12 months? [__][__]

2.2 Did you attend any training session for this intervention? [__] (1=Yes; 2=No)

If yes, how many training sessions did you attend during the last 12 months? [__][__]

2.3 Could you please kindly assist to complete the following table for me?

**Table 2.3 Training costs**

| **Training sessions** | **Funded by**  1. Community  2. Individuals  3. Local NGO  4. International NGO  5. Government  6. UN agencies | **Total per diem** | **Total travel cost** | **Other costs** | **Specify type of “other costs”** |
| --- | --- | --- | --- | --- | --- |
| **a. Conducted** |  |  |  |  |  |
|  |  |  |  |  |  |
|  |  |  |  |  |  |
|  |  |  |  |  |  |
|  |  |  |  |  |  |
|  |  |  |  |  |  |
|  |  |  |  |  |  |
| **b. Attended** |  |  |  |  |  |
|  |  |  |  |  |  |
|  |  |  |  |  |  |
|  |  |  |  |  |  |
|  |  |  |  |  |  |
|  |  |  |  |  |  |
|  |  |  |  |  |  |

**3. Recurrent Social Mobilization Costs**

3.1 Did you conduct any social mobilization for this intervention? [__] (1=Yes; 2=No)

3.2 If yes, how many times during the last 12 months? [__][__]

3.3 Could you please kindly assist to complete the following table?

**Table 3.3 Social mobilisation**

| **List social mobilization conducted** | **Funded by**  1. Community  2. Individuals  3. Local NGO  4. International NGO  5. Government  6. UN agencies | **Total per diem spent** | **Total travel cost** | **Cost of IEC material** | **Other costs** | **Specify type of “other costs”** |
| --- | --- | --- | --- | --- | --- | --- |
|  |  |  |  |  |  |  |
|  |  |  |  |  |  |  |
|  |  |  |  |  |  |  |
|  |  |  |  |  |  |  |
|  |  |  |  |  |  |  |
|  |  |  |  |  |  |  |
|  |  |  |  |  |  |  |
|  |  |  |  |  |  |  |
|  |  |  |  |  |  |  |
|  |  |  |  |  |  |  |
|  |  |  |  |  |  |  |
|  |  |  |  |  |  |  |
|  |  |  |  |  |  |  |

**4 Vehicles, motor cycles and bicycles used during the year for the interventions**

Please give information about the following transportation assets/equipment used for various programmes.

**5. Recurrent maintenance and utilities costs**

It is important for our study to know what the expenditures for maintenance and utilities were for your programme. Could you please assist to complete the following table for the maintenance and utilities cost for your programme during the last 12 months?

**Table 5.1 Maintenance**

| **Maintenance/Utility Item** | **Amount spent during the last 12 months** |
| --- | --- |
| 1. Fuel (vehicle, generator) |  |
| 1. Maintenance (vehicle and generator) |  |
| 1. Telephone |  |
| 1. Vehicle insurance |  |
| 1. Other (specify) |  |
|  |  |

**6. Recurrent Supervision and Monitoring Costs**

6.1 Did you conduct any supervision and monitoring for this intervention? [__] (1=Yes; 2=No)

6.2 If yes, how many times did you do it during the last 12 months? [__][__]

6.3 Could you please kindly assist to complete the following table?

**Table 6.1 Supervision and Monitoring Costs**

| List the number of supervision/monitoring activities | Funded by  1. Community  2. Individuals  3. Local NGO  4. International NGO  5. Government  6. UN agencies | Total per diem spent | Total transport cost (exclude use of office vehicle) | Total other costs | Specify type of “other costs” |
| --- | --- | --- | --- | --- | --- |
|  |  |  |  |  |  |
|  |  |  |  |  |  |
|  |  |  |  |  |  |
|  |  |  |  |  |  |
|  |  |  |  |  |  |

This completes the interview. Thank you very much for your time and for providing us with the information.

**Interviewer:** Name: ________________________ Signature: _____________________

**Project economist:** Form checked [__] (1=Yes; 2=No) Signature: _________________

**Instructions for completing Instrument 14**

Questionnaire for Programme Managers at Institutional Level

Person to be interviewed: Programme Managers

Programme Manager: Senior personnel responsible for each of the following programmes: Long Lasting Insecticide Treated Nets (LLIN); Home Management of Malaria (HMM), Vitamin A (Vit-A) and Praziquantel at all institutional levels.

**Project Code**

Country: Malawi Project Code: 1

**District Code**

District: Mangochi District Code: 1

District: Mzimba District Code: 2

**Interventions**

HMM = 1

LLIN = 2

Vit-A = 3

Schistosomiasis = 4

Control = 6

**Q1: Recurrent Personnel Cost** (recurrent inputs are resources that are expected to be consumed (or replaced) within a year. These include monetary payment to personnel such as managers, physicians, nurses, supervisors, health workers, administrators, technicians, consultants, CDI implementers, community leaders, drivers, casual labour.

**Table 1.2.a**

1. **Salaried Employees:** All personnel employed by the institutions working for either or all of the above programmes, being paid monthly salaries. Please list each of them, one person per row. Do not fill names of individuals.
2. **Category:** This refers to office designations, such as:
   1. Programme Manager/Coordinator
   2. Assistant Manager
   3. Medical Officer
   4. Technician
   5. Nurse
   6. Secretary
   7. Drivers
   8. Other, specify.
3. **Grade:** This refers to salary scale schedule for specific salaried personnel. Refer to local salary grade for specific study site. Code as mentioned by the respondent.
4. **Monthly Gross Salary:** This includes the total monthly pay package before tax is deducted. Use the national currency.
5. **Funded by:** Agents responsible for the financing of the cost of the personnel/programme or activities. Please use the following codes:
6. Community
7. Individuals
8. Local NGO
9. International NGO
10. Government
11. UN agencies
12. **Additional Allowances:** Refers to annual additional allowances provided for the study intervention and that are not included in the monthly salary.
13. **Could you estimate the average number of days per year and hours per day you allocated to the following intervention?** Please ask the respondent to provide an estimate the number of days per year and number of hours per day she or he spent during the year for the study intervention for which the respondent is responsible.

**Table 1.2.b**

1. **Volunteers:** This refers to personnel working on the intervention, but without receiving a monthly salary. Please list each of them, one person per row. Do not fill names of individuals. Do not include volunteers that work at community level, such as CDI Implementers, as these will be covered elsewhere.
2. **Education:** This refers to level of education of the volunteer:
3. Tertiary education
4. Secondary education
5. Primary or less
6. **Type of volunteer work:** Specific activities or job done or carried out by the volunteer in the course of the intervention. Please use the following codes:
7. Administrative
8. Technical assistance
9. Supplies
10. Driver
11. Others, specify
12. **Additional Allowances:** As defined in table 1.2.a.
13. **Funded by:** As defined in table 1.2.a.
14. **Could you estimate the average number of days per year and hours per day you allocated to the following intervention?** Please ask the respondent to provide an estimate the number of days per year and number of hours per day she or he spent during the year for the study intervention for which the respondent is responsible.

**Table 1.2.c**

1. **Consultants:** Specialized individual or organization providing technical service in relation to the intervention. Please list each of them, one person per row. Do not fill names of individuals.
2. **Type of consultancy:**
3. Management consultant
4. Public health specialist
5. Other technical expertise
6. Transport
7. Other, specify
8. **Fees and other Allowances: (Annual cost to be collected)** Monetary payment to the services rendered by the consultant.
9. **Funded by:** As defined in table 1.2.a.

**Q2: Recurrent Training Cost:** These are payments for short-term training programmes (in less than a year). They include expenditures on transport, training fees, stationeries, photocopies, telephone charges, per diems etc.

2.1 Probe for Yes or No response to whether training session related to intervention activities was conducted.

If yes, in 2.1, specify the number of sessions conducted.

2.2 Probe for Yes or No response to whether training session related to intervention activities was attended.

If yes, in 2.2, specify the number of training sessions attended.

**2.3 Table 2.3.**

1. **Training Sessions:** This requires listing the type of training that was conducted or attended by the respondent.
2. **Funded by:** Agents responsible for the financing of the training. Please use the following codes:
3. Community
4. Individuals
5. Local NGO
6. International NGO
7. Government
8. UN agencies
9. **Total per diem:** This refers to total amount paid for accommodation, feeding and other incidences relating to the training session.
10. **Total Travel Cost:** This includes all travel related expenses, excluding the use of official/programme vehicles e.g. flight, water transport, road transport.
11. **Total Other Cost:** All other costs not captured above e.g. stationeries, photocopies etc.
12. **Specify type of “other costs”:** Provide a corresponding description of cost entered in the preceding column.

**Q3: Recurrent Social Mobilization:** Are variable expenditures on various mobilization activities carried out for the intervention. Such expenditures would involve items such as travels, telephone charges, communication materials, radio/TV or newspaper advertisements etc.

3.1 Probe for Yes or No response to whether mobilization related to the intervention activities was conducted.

3.2 Follow up question to Yes response in 3.3 for number of activities.

**3.3 Table 3.3**

1. **List Social Mobilization Conducted:** Enter specific list of the intervention mobilization activities conducted.
2. **Funded by:** Agents responsible for the financing of the activity. Please use the following codes:
3. Community
4. Individuals
5. Local NGO
6. International NGO
7. Government
8. UN agencies
9. **Total per diem:** This refers to total amount paid for accommodation, feeding and other incidences relating to the training session.
10. **Total Travel Cost:** This includes all travel related expenses, excluding the use of official/programme vehicles e.g. flight, water transport, road transport.
11. **Cost of IEC Material:** Request for total amount of money used to develop communication materials for mobilization activities.
12. **Total Other Cost:** All other costs not captured above e.g. stationeries, photocopies etc.
13. **Specify type of “other costs”:** Provide a corresponding description of cost entered in the preceding column.

**Table 4**

1. **Type:** Name the type of the vehicle, motorcycle or bicycle used for the intervention
2. **Quantity:** Give amount/number of vehicles, motorcycles or bicycles used as listed in ‘Type’ column.
3. **Specify Make/Model No:** Write the brand name of the equipment and its model number.
4. **Purchased/Rented or Donated:** Respondent to indicate how the equipment was acquired.
5. **Purchased/Donated by:** If purchased or donated, please indicate who or which organization purchased/donated the equipment using the following codes:
6. Community
7. Individuals
8. Local NGO
9. International NGO
10. Government
11. UN agencies
12. **Do other programmes use this equipment:** Probe for a Yes or No response for the assistance of other programmes using the same equipment. Fill in the corresponding number relating to Yes or No.
13. **Could you estimate the average number of days per year and hours per day you allocated to the following intervention?** Please ask the respondent to provide an estimate the number of days per year and number of hours per day she or he spent during the year for the study intervention for which the respondent is responsible.

**5. Recurrent Maintenance and Utility Costs**

Definition: This includes operational cost such as electricity, water, heating, fuel, telephone, fax, telex, insurance, cleaning, painting, repairs of electricity, plumbing, roofing and heating, fuel, vehicle services.

**Table 5.1** Request for monetary figures (in local currency) for each item listed. Figure for some of these items may exist in the books/records of the institution.

**6. Current Supervision and Monitoring Costs**

Definition: This includes all expenses incurred in facilitating the supervision and monitoring of the intervention. Such expenses may be incurred on such items as accommodation, feeding allowance, travel expenses and other incidences.

6.1 Probe for Yes or No response to whether supervision and monitoring activities were carried out.

6.2 Follow up question to Yes response in 6.1 for number of times supervision and monitoring activities were carried out.

**6.3 Table 6.1**

1. **List number of supervision and monitoring activities conducted:** Enter specific list of the supervision and monitoring activities for the intervention conducted
2. **Funded by:** Agents responsible for the financing of the activity. Please use the following codes:
3. Community
4. Individuals
5. Local NGO
6. International NGO
7. Government
8. UN agencies
9. **Total per diem:** This refers to total amount paid for accommodation, feeding and other incidences relating to the training session.
10. **Total Travel Cost:** This includes all travel related expenses, excluding the use of official/programme vehicles e.g. flight, water transport, road transport.
11. **Total Other Cost:** All other costs not captured above e.g. stationeries, photocopies etc.
12. **Specify type of “other costs”:** Provide a corresponding description of cost entered in the preceding column.

b. Give information on the unit cost of drug/LLIN and the transport cost incurred on drugs received in the last 12 months

| S/N | DRUGS/LLIN | Type | Current Unit Price (To be filled by Economist) | Cost of Transportation |
| --- | --- | --- | --- | --- |
|  | Antimalarial tablets | Artesunate |  |  |
|  |  | Coartem |  |  |
|  |  | ACT |  |  |
|  |  | Other |  |  |
|  | LLIN (regular) units | Large |  |  |
|  |  | Small |  |  |
|  | LLIN (long lasting) units | Large |  |  |
|  |  | Small |  |  |
|  | LLIN Retreatment Kits | Tablet |  |  |
|  |  | Sachet |  |  |
|  |  | Liquid |  |  |
|  | Vitamin A | Capsule |  |  |
|  | Praziquantel | Tablet |  |  |

| **FORM 12: PERSONNEL AND OTHER RUNNING COST - COST INSTRUMENT FOR FIRST LINE HEALTH FACILITY AT END OF YEAR** |
| --- |

**IDENTITY NUMBER (IDNO): ______ (e.g. MAL01, the first 3 digits represent the study site while the last 2 digits represent the serial number of the interview)**

**PROJECT CODE [__] DISTRICT CODE [__]**

**DATE [__][__]/[__][__]/[__][__][__][__]**

**EVALUATION [__] (0=Baseline; 1=Evaluation)**

**INTERVIEW OF OFFICER IN-CHARGE OF FLHF**

**[__] HMM [__] LLIN [__] VITAMIN A [__] SCHISTO**

**The following questionnaire is to be administered at the First Line Health Facility (≤50 that is 1 FLHF Staff per community where applicable).**

**1. Recurrent Personnel Costs**

1.1 Can you tell me how many personnel are involved in this intervention?

1. Number of salaried employees? [__][__]
2. Number of volunteers at the level of your institution? [__][__]
3. Number of consultants? [__][__]

*Please include all personnel involved, whether they work for the government or for other organizations e.g. NGOs (but do not include community level volunteers)*

**2. Recurrent Training Costs**

2.1 Did you conduct any training session for any of the following?

1. HMM [__] (1=Yes; 2=No)
2. LLIN [__] (1=Yes; 2=No)
3. Vitamin A [__] (1=Yes; 2=No)
4. Schistosomiasis [__] (1=Yes; 2=No)

2.2 If yes, how many training sessions did you conduct during the 12 months for the following?

Number of sessions

1. HMM [__][__]
2. LLIN [__][__]
3. Vitamin A [__][__]
4. Schistosomiasis [__][__]

2.3 Did you attend any training session for any of the following?

1. HMM [__] (1=Yes; 2=No)
2. LLIN [__] (1=Yes; 2=No)
3. Vitamin A [__] (1=Yes; 2=No)
4. Schistosomiasis [__] (1=Yes; 2=No)

2.4 If yes, how many training sessions did you attend during the last 12 months for the following?

Number of sessions

1. HMM [__][__]
2. LLIN [__][__]
3. Vitamin A [__][__]
4. Schistosomiasis [__][__]

2.5 Could you assist to complete the following table for me?

**Table 2.5 Training Sessions**

| **Training sessions** | Funded by *(code as many as relevant)*  1. Community  2. Individuals  3. Local NGO  4. International NGO  5. Government  6. UN agencies | **Total allowance spent/received** | **Transportation cost** | **Other costs** | **Specify type of “other costs”** |
| --- | --- | --- | --- | --- | --- |
| **a. Conducted** |  |  |  |  |  |
|  |  |  |  |  |  |
|  |  |  |  |  |  |
|  |  |  |  |  |  |
|  |  |  |  |  |  |
|  |  |  |  |  |  |
|  |  |  |  |  |  |
| **b. Attended** |  |  |  |  |  |
|  |  |  |  |  |  |
|  |  |  |  |  |  |
|  |  |  |  |  |  |
|  |  |  |  |  |  |
|  |  |  |  |  |  |
|  |  |  |  |  |  |

**3. Recurrent Social Mobilization Costs**

3.1 Did you conduct social mobilization for any of the following?

1. HMM [__] (1=Yes; 2=No)
2. LLIN [__] (1=Yes; 2=No)
3. Vitamin A [__] (1=Yes; 2=No)
4. Schistosomiasis [__] (1=Yes; 2=No)

3.2 If yes, how many times during the last 12 months?

Number of times of social mobilizations

1. HMM [__][__]
2. LLIN [__][__]
3. Vitamin A [__][__]
4. Schistosomiasis [__][__]

3.3 Could you please assist to complete the following table?

**Table 3.3 Social Mobilization**

| **List Social Mobilization conducted** | Funded by *(code as many as relevant)*  1. Community  2. Individuals  3. Local NGO  4. International NGO  5. Government  6. UN agencies | **Total allowance spent** | **Transportation cost** | **Other costs** | **Specify type of “other costs”** | **Total cost** |
| --- | --- | --- | --- | --- | --- | --- |
|  |  |  |  |  |  |  |
|  |  |  |  |  |  |  |
|  |  |  |  |  |  |  |
|  |  |  |  |  |  |  |
|  |  |  |  |  |  |  |
|  |  |  |  |  |  |  |
|  |  |  |  |  |  |  |

**5. Recurrent maintenance and utilities costs**

5.1 It is important for our study to know what the expenditures for maintenance and utilities were for your first line health facility. Could you please assist to complete the following table for the maintenance and utilities of your FLHF during the last 12 months?

**Table 5.1 Maintenance**

| **Maintenance/Utility Item** | **Amount spent during the last 12 months** |
| --- | --- |
| 1. Fuel (vehicle, motorcycle and generator) |  |
| 1. Maintenance (vehicle, motorcycle and generator) |  |
| 1. Telephone |  |
| 1. Vehicle, motorcycle insurance |  |
| 1. Other (specify ………………………) |  |
|  |  |

**6. Recurrent Supervision and Monitoring Costs**

6.1 Did you conduct any supervision and monitoring for the following?

1. HMM [__] (1=Yes; 2=No)
2. LLIN [__] (1=Yes; 2=No)
3. Vitamin A [__] (1=Yes; 2=No)
4. Schistosomiasis [__] (1=Yes; 2=No)

6.2 If yes, how many times did you do it within the last 12 months?

Number of supervision and monitoring

1. HMM [__][__]
2. LLIN [__][__]
3. Vitamin A [__][__]
4. Schistosomiasis [__][__]

6.3 Could you please assist to complete the following table?

**Table 6.3 Supervision and Monitoring Activities**

| List the number of supervision /monitoring activities | Funded by *(code as many as relevant)*  1. Community  2. Individuals  3. Local NGO  4. International NGO  5. Government  6. UN agencies | Total allowance spent | Total transport cost (exclude use of office vehicle or motorcycle) | Total other costs | Specify type of “other costs” |
| --- | --- | --- | --- | --- | --- |
|  |  |  |  |  |  |
|  |  |  |  |  |  |
|  |  |  |  |  |  |
|  |  |  |  |  |  |
|  |  |  |  |  |  |
|  |  |  |  |  |  |

***This completes the interview. Thank you very much for you time and for providing us with the information.***

**Interviewer:** Name ___________________________ Signature _________________

**Project economist:** Form checked [__] (1=Yes; 2=No) Signature _________________

1.2 Give information on the unit cost of drug/LLIN and the transport cost incurred on drugs received in the last 12 months

| S/N | DRUGS/LLIN | Type | Current Unit Price (To be filled by Economist) | Cost of Transportation |
| --- | --- | --- | --- | --- |
|  | Antimalarial tablets | Artesunate |  |  |
|  |  | Coartem |  |  |
|  |  | ACT |  |  |
|  |  | Other |  |  |
|  | LLIN (regular) units | Large |  |  |
|  |  | Small |  |  |
|  | LLIN (long lasting) units | Large |  |  |
|  |  | Small |  |  |
|  | LLIN Retreatment Kits | Tablet |  |  |
|  |  | Sachet |  |  |
|  |  | Liquid |  |  |
|  | Vitamin A | Capsule |  |  |
|  | Praziquantel | Tablet |  |  |

| **FORM 13: QUESTIONNAIRE FOR COMMUNITY LEADERS/KEY INFORMANT AT THE COMMUNITY LEVEL TO OBTAIN COST INFORMATION AT END OF YEAR** |
| --- |

**Project code [__] District code [__] Village code [__][__]**

**Date [__][__]/[__][__]/[__][__][__][__] Evaluation [__] (0=Baseline; 1=Evaluation)**

**Interview of community leaders**

**[__] HMM [__] LLIN [__] Vitamin A [__] Schistosomiasis**

***(Where necessary check information with Medical Personnel in Charge of the Community Health Unit/Centre).* One leader per community. Total: 50 per site.**

The Community-directed Intervention intends to use the Community as it is used for delivery of health interventions (LLINs, Home Management of Malaria, Vitamin A and Praziquantel). In this context, the Community, the Health Services and other partners have specific sites to perform in order to ensure the integration of these interventions.

Please would you like to take part and provide information to the following questions? Information provided will be kept strictly confidential.

[If yes, proceed with interview. If no, Stop]

**A. Socio-demographic information**

Sex [__] (1=Male; 2=Female)

Number of years of experience [__][__]

Designation __________________________________

1. Could you kindly tell me how these programmes are organized in this community? *(Mention the relevant interventions [HMM, LLIN, Vitamin A, Schistosomiasis] for the community and note all that is said).*

HMM: __________________________________________________________________

________________________________________________________________________

________________________________________________________________________

LLIN: ___________________________________________________________________

________________________________________________________________________

________________________________________________________________________

VIT A: _________________________________________________________________

________________________________________________________________________

________________________________________________________________________

PRAZIQUANTEL: _______________________________________________________________

________________________________________________________________________

________________________________________________________________________

2. How many CDI Implementers are involved in these interventions in your community? [__][__]

**3. Antimalarial drugs for home management of malaria.**

3.1 From where does the community get antimalarial drugs?

1. Community [__]
2. Individuals [__]
3. Local NGO [__]
4. International NGO [__]
5. Health facility [__]
6. UN agencies [__]

3.2 Does your community incur any cost to purchase drugs from the health facility? [__] 1=Yes; 2=No

3.3 If yes, how much did your community spend to purchase antimalarial drugs from the health facility within the last 12 months? *(Please check available records)*

Cost of drugs: __________________

3.4 How many packages/tablets did you purchase during the last 12 months from the health facility?

1. For children __________________
2. For adults ____________________

**4. Praziquantel drugs for treatment of schistosomiasis.**

4.1 From where does the community get schistosomiasis drugs?

1. Community [__]
2. Individuals [__]
3. Local NGO [__]
4. International NGO [__]
5. Health facility [__]
6. UN agencies [__]

4.2 Does your community incur any cost to purchase drugs from the health facility? [__] 1=Yes; 2=No

4.3 If yes, how much did your community spend to purchase schistosomiasis drugs from the health facility within the last 12 months? *(Please check available records)*

Cost of drugs: __________________

4.4 How many tablets did you purchase during the last 12 months from the health facility?

Number of tablets __________________

**5. Insecticide treated nets**

5.1 From where does the community get insecticide treated nets?

1. Community [__]
2. Individuals [__]
3. Local NGO [__]
4. International NGO [__]
5. Health facility [__]
6. UN agencies [__]

5.2 Does your community incur any costs to purchase insecticide treated nets or retreatment kits from the health facility or from an NGO?

1. LLINs [__] 1=Yes; 2=No
2. LLIN Retreatment Kits [__] 1=Yes; 2=No

5.3 If yes, how many nets or retreatment kits did your community purchase from the health facility or from an NGO within the last 12 months?

Quantity

1. LLINs (regular) _________________
2. LLINs (long lasting) _________________
3. LLIN Retreatment Kits _________________

5.4 If yes, how much did your community spend to purchase the following materials from the health facility within the last 12 months? (Please check available records).

Amount

1. LLINs (regular) _________________
2. LLINs (long lasting) _________________
3. LLIN Retreatment Kits _________________

**6. Recording materials and stationeries**

6.1 Does your community incur any costs on recording materials and stationeries for documenting activities related to the following interventions in this community?

1. HMM [__] 1=Yes; 2=No
2. LLINs [__] 1=Yes; 2=No
3. Vitamin A [__] 1=Yes; 2=No
4. Praziquantel [__] 1=Yes; 2=No

6.2 If yes, how much did your community spend on recording materials and stationeries for CDI activities during the last 12 months?

Cost of recording materials

1. HMM _______________
2. LLINs _______________
3. Vitamin A _______________
4. Praziquantel _______________

**7. Capital inputs**

7.1 Are there any buildings in the village that are used for the study interventions?

1. HMM [__] 1=Yes; 2=No
2. LLINs [__] 1=Yes; 2=No
3. Vitamin A [__] 1=Yes; 2=No
4. Schisto [__] 1=Yes; 2=No

7.2 If yes, please describe what kind of buildings and for what they are used? *(Listen to the description and code as follows :)* [__]

1=Wood and bamboo

2=Burnt bricks

3=Cement bricks

4=Mud

**Instructions for completing instruments 17 for community leaders**

The questionnaire is to be administered to Community Leaders and Volunteers (where necessary check information with medical personnel in charge of the community health unit/centre).

Q1. Allow the community leader to describe what goes on with the intervention in the community, Take note of all the points mentioned.

Q2. Let the community leader tell you how many volunteers are involved in the interventions if this is not already mentioned in Q1 above.

Q3. ANTIMALARIAL DRUGS FOR HOME MANAGEMENT OF MALARIA

3.1 This refers to where the drugs came from:

1. Community
2. Individuals
3. Local NGO
4. International NGO
5. Health facility
6. UN agencies

3.2 The cost refers to purchased cost for the children and adult drugs

3.3 This refers to the total amount of drugs the community have made use of during the year

Q4. PRAZIQUANTEL DRUGS FOR TREATMENT OF SCHISTOSOMIASIS

4.1 This refers to where the drugs came from:

1. Community
2. Individuals
3. Local NGO
4. International NGO
5. Health facility
6. UN agencies

4.2 The cost refers to purchased cost for the drugs

4.3 This refers to the total amount of drugs the community have made use of during the year

Q5. LONG LASTING INSECTICIDE TREATED NETS

- 1. This refers to the sources of where the LLINs/retreatment kits came from and the following codes are to be used:

1. Community
2. Individuals
3. Local NGO
4. International NGO
5. Health facility
6. UN agencies

5.2 The cost refers to the purchased cost of LLIN and retreatment kits if they were purchased only

5.3 This is the total amount of LLIN and retreatment kits the community used during the year.

Q6. RECORDING MATERIALS AND STATINERIES: This is the total amount used on materials and stationeries by the community for these interventions.

Q7.1.1 Buildings: *(for information and not for costing).* The question is to elicit a yes or no response for formal buildings specifically used for the interventions. Formal buildings are buildings specifically designated for interventions (excluding domestic houses).

Q7.1.2 These are materials used for constructing the formal building, the following codes are to be used:

1. Wood and bamboo
2. Burnt bricks
3. Cement bricks
4. Mud

Q7.2.1 Equipment. These

**Item:** These are already listed. However others could be included as relevant.

**How many:** Give quantity of each equipment type used for the relevant intervention.

**Specify Make/Model No:** Write the brand name of the equipment and its model number.

**Cost (To be filled later):** This should not be completed during interview. The economist will later fill this in.

**Purchased/Rented or Donated:** Respondent to indicate how the equipment was acquired. Fill in the corresponding number.

**Who purchased/rented/donated it:** Respondent to give the name of a person/organization responsible for buying or donating the listed equipment.

**Do other programmes use this equipment:** Probe for a Yes or No response for the assistance of other programmes using the same equipment. Fill in the corresponding number relating to Yes or No.

Q7.2.2 Transport and assets. These

**Type:** Name the type of the vehicle, motorcycle or bicycle used for intervention.

**Quantity:** Give amount/number of vehicles, motorcycles or bicycles used as listed in the ‘Type’ column.

**Specify Make/Model No:** Write the brand name and model number of the transportation asset.

**Cost (To be filled later):** This should not be completed during interview. The economist will later fill this in.

**Purchased/Rented or Donated:** Respondent to indicate how the transportation asset was acquired. Fill in the corresponding number.

**Name the person/organization responsible for purchasing the vehicle:** Respondent to give the name of the person/organization responsible for buying or donating the listed transportation asset.

**Do other programmes use this transportation asset:** Probe for a Yes or No response for the assistance of other programmes using the transportation asset. Fill in the corresponding number relating to Yes or No.

**Could you estimate the average number of days per year and hours per day you allocated to the following interventions?** Please ask the respondent to provide an estimate the average number of days she or he spent during the year for the study intervention for which the respondent is responsible.

| **FORM 14: QUESTIONNAIRE FOR VOLUNTEERS AT END OF YEAR** |
| --- |

**PROJECT CODE [__] DISTRICT CODE [__] VILLAGE CODE [__][__]**

**DATE [__][__]/[__][__]/[__][__][__][__] EVALUATION [__] (0=Baseline; 1=Evaluation)**

**INTERVIEW OF VOLUNTEERS [__] HMM [__] LLIN [__] VITAMIN A [__] SCHISTO**

***(Where necessary check information with Medical Personnel in Charge of the Community Health Unit/Centre).* Interview between 1 and 5 volunteers per community ensuring that all the interventions are covered.**

Did you attend any training session in the last 12 months? [__] (1=Yes; 2=No)

If yes, how many? _________________

Could you give details of the training sessions you attended in the following table?

**Table 2: Training Sessions**

| **Training sessions attended** | **Total allowance received** | **Funded by *(code as many as relevant)***  1. Community  2. Individuals  3. Local NGO  4. International NGO  5. Government  6. UN agencies | **Total transportation cost** | **Total other costs** | **Specify type of “other costs”** |
| --- | --- | --- | --- | --- | --- |
|  |  |  |  |  |  |
|  |  |  |  |  |  |
|  |  |  |  |  |  |
|  |  |  |  |  |  |
|  |  |  |  |  |  |
|  |  |  |  |  |  |
|  |  |  |  |  |  |

**Instructions for completing instruments 14 for volunteers**

Table 1

**S/N:** Serial number for the person interviewed in the order of the interviews

**Profession** refers to the main occupation or the primary income earning activity of the individual. Follow the example and write in the occupation. Later, this should be coded as follows

1. Farmer
2. Teacher
3. Other profession
4. Retired civil servant
5. Student
6. Unemployed
7. Other (specify)

**Grade level** is the level that the individual is in the formal primary duty he is/was employed.

To be used as given by respondent but converted later according to national grade by the health economist.

**Monthly gross salary** includes total monthly pay package before tax is deducted.

**The type of volunteer activities** is the duties of the individual in the community to be coded as follows:

- - - 1. CDI Implementer
      2. HMM role mothers
      3. LLIN retreatment
      4. Local guide
      5. Other (specify)

**Number of days** refers to the total number of days spent by the volunteer on the intervention by year.

**Number of hours per day** refers to the total number of hours spent per day by the volunteer on the intervention.

**Allowances and other financial incentives for volunteer activity per year** refer to the totality of payment, which is not part of the main gross monthly salary in column 4.

**Funded by** refers to who is responsible for these allowances. This is coded as the following

1. Community
2. Individual
3. Local NGO
4. International NGO
5. Government
6. UN agencies

**Other benefits** are the material incentives given to the volunteer with the following codes

1. Meals
2. Yam
3. Rice
4. Beans
5. Plantain
6. Maize
7. Other (specify)

**Quantity** is to be expressed in local measurement in the following codes

1. Pieces
2. Bag
3. Cup
4. Kg
5. Others specify

**Benefits funded** by refer to the provider of the other benefits. This is coded as the following

1. Community
2. Individual
3. Local NGO
4. International NGO
5. Government
6. UN agencies

**Trainings**

**Training sessions:** This requires listing the type of training that was conducted or attended by the respondent and the number of days the respondent attended

**Total Allowance received:** This refers to all monies the volunteer received

**Funded by:** Agents responsible for the financing of the training. Please use the following codes:

1. Community
2. Individual
3. Local NGO
4. International NGO
5. Government
6. UN agencies

**Total transportation cost:** This includes all travel related expenses to and from the training sites.

**Total other costs:** This refers to other expenditures the volunteer made in the course of attending the training for skills related to the implementation of the study intervention(s).

| **FORM 15: QUESTIONNAIRE FOR HOUSEHOLD - TO OBTAIN COST INFORMATION AT END OF YEAR** |
| --- |

**PROJECT CODE [__] DISTRICT CODE [__] VILLAGE CODE [__][__]**

**HOUSEHOLD NUMBER [__][__][__] DATE [__][__]/[__][__]/[__][__][__][__]**

**EVALUATION [__]** (0=Baseline 1=Evaluation)

**INTERVIEW OF VOLUNTEERS** [__] HMM [__] LLIN [__] Vitamin A [__] Schisto

***(Data to be collected during coverage surveys and in the same households that are included in the coverage survey)***

**Introduction**

Good day, I am ________________ from the Ministry of Health and wish to learn about your participation in the delivery of Community-directed interventions such as Vitamin A distribution, Home management of malaria (HMM), Long lasting insecticide treated nets (LLIN) and Praziquantel that are going on in this community. I am working for study team based in Lilongwe. The team is investigating the possibility of delivering health interventions in the community through Community directed interventions (CDI) approach.

The Community-Directed Interventions strategy intends to use the community for delivery of other interventions such as LLINs, Home management of malaria and vitamin A distribution. In this context, the community, the health services and other partners have specific roles to perform in order to ensure the integration of these interventions.

We will ask you questions about your personal experiences. The questions are general but if you find that some questions are not going well with you, please do not feel compelled to answer any of them for any reason. We will talk to you for about 10 -15 minutes. Participation in this interview is voluntary and you may choose to terminate the interview if you decide without any repercussion. What you tell us will help us develop a strategy for training community members to manage the interventions stated earlier and thus improve the health of the people in this community. Your name and what you say to us for this study will be kept private.

Do you have any questions about the study? If you have any questions about your rights in the study or in case of emergency, you may contact Mr Peter Makaula of RHED-Malawi on +265 888 850 829.

Are you willing to participate? Yes [__] No [__]

**If no, thank participants and terminate discussion.**

1. Did you or any member of your household attend any community meeting for any of the following interventions during the last 12 months?

*Probe for:* 1=Yes, 2=No

1. HMM [__]
2. LLIN [__]
3. Vitamin A [__]
4. Schistosomiasis [__]

*Probe further to differentiate between mobilization meetings for any of the above interventions and other community meetings not related to these interventions*

1. How many community mobilization meetings did you or any member of your household attend during the last 12 months?

*Probe for:* Number of community mobilization meetings

1. HMM [__][__]
2. LLIN [__][__]
3. Vitamin A [__][__]
4. Schisto [__][__]

*Make sure to exclude mobilization meetings that are not related to the study interventions*

1. How many adults (15 years and above) attended any of these meetings from your household? [__][__]
2. Can you tell me how many meeting each of them attended and how much time they spend in attending and travelling to and from the meeting(s)?

| Household member no. | Number of meetings attended | Kindly estimate the total time spent in hours attending these meetings. Please include the travel time to and from the meeting |
| --- | --- | --- |
|  |  |  |
|  |  |  |
|  |  |  |
|  |  |  |
|  |  |  |
|  |  |  |
|  |  |  |
|  |  |  |
|  |  |  |
